# Inhibiting Postoperative Fibrosis in Glaucoma Filtration Surgery Through Porous PLLA/RGD Membrane Mediated Local/Sustained Delivery of Mitomycin C

**DOI:** 10.1167/tvst.14.12.1

**Published:** 2025-12-01

**Authors:** Xia Wu, Kaili Wu, Shibing Ni, Shiyi Song, Dadong Jia, Junjie Tang, Jiabing Ran, Liang Liang

**Affiliations:** 1 Department of Ophthalmology, The Second People's Hospital of China Three Gorges University, The Second People's Hospital of Yichang, Hubei, People's Republic of China; 2 Zhongshan Ophthalmic Center, State Key Laboratory of Ophthalmology, Sun Yat-Sen University, Guangdong Provincial Clinical Research Center for Ocular Diseases, Guangzhou, People's Republic of China; 3 College of Materials and Chemical Engineering, Key Laboratory of Inorganic Nonmetallic Crystalline and Energy Conversion Materials, China Three Gorges University, Yichang, People's Republic of China; 4 Hubei Institute Geological Prospecting Equipment, Wuhan, People's Republic of China; 5 College of Biological and Pharmaceutical Sciences, China Three Gorges University, Yichang, People's Republic of China

**Keywords:** glaucoma, electrospinning, mitomycin C (MMC)

## Abstract

**Purpose:**

The purpose of this study was to investigate the characteristics of sustained drug release systems established by polylactic acid (PLLA)/RGD/mitomycin C (MMC) electrospun nanofiber membrane in vitro, and confirm its anti-scarring effect in a rabbit model of glaucoma filtration surgery (GFS).

**Methods:**

In vitro experiments: (1) PLLA/RGD/MMC nanofiber membrane drug delivery system was prepared by electrospinning technology. (2) Characterization of nanofiber membrane. (3) Biocompatibility detection of nanofiber membrane. In vivo experiment: construct a surgical model for rabbit eye GFS, divided into four groups: the control group, the PLLA/RGD membrane group, the 0.4 mg/mL MMC group, and the PLLA/RGD/MMC membrane group. The morphology of filtering blebs and wound healing were observed on the 7th, 14th, and 28th days after the operation. At 28 days after the operation, histological and immunohistochemical staining were performed to observe the scar formation. Finally, the results were statistically analyzed.

**Results:**

The composition, structure, hydrophily, thermal behaviors, degradability, and mechanical properties of the membrane were investigated in detail. In vitro cell culture assay indicated that the PLLA/RGD/MMC2 membrane could release MMC in a sustained manner for over 25 days. In vitro cell culture assay proved the superior cytocompatibility of the membrane with Human embryonic Tenon’s capsule fibroblasts (HFTFs). Histology and immunohistochemistry indicated that the membrane could efficiently inhibit scaring formation after GFS and showed significant advantage over the conventional MMC cotton pad.

**Conclusions:**

The as-prepared PLLA/RGD/MMC membrane could be a potential candidate for inhibiting scaring formation after GFS in the future.

**Translational Relevance:**

This work shed some light to the developments and applications of MMC loaded electrospinning membrane for inhibiting postoperative fibrosis in GFS.

## Introduction

Glaucoma, a severe eye disease, featuring unexplained optic nerve damage and subsequent alterations in visual function, has become the leading cause of irreversible blindness globally.^1^ Because optic nerve damage is mainly caused by pathological high intraocular pressure (IOP), reducing IOP has been recognized as one of the most effective strategies for glaucoma treatment.^2^ In the clinic, glaucoma filtration surgery (GFS) is a commonly used surgical method when drugs and laser treatments cannot control IOP, but postoperative scarring in the filtration channel often leads to surgical failure.^3^^,^^4^ In the past decades, the number of patients with glaucoma has been on the rise and estimated to be over 112 million in 2040.^5^ Therefore, exploring advanced techniques or materials to inhibit postoperative fibrosis in GFS is of vital importance and has drawn tremendous attention.

Mitomycin C (MMC), an efficient anti-fibrosis drug produced by *Streptomyces caespitosus*, has proved to be capable of suppressing postoperative scar formation.^6^ Although the rationale behind MMC restricting fibrogenesis has not been clearly elucidated, it is widely believed to be related to disturbed metabolization of fibroblasts.^7^ Gray et al. reported that MMC prevented long-term proliferation of fibroblasts through blocking DNA synthesis.^8^ In addition, it has also been found that MMC could affect expression of certain miRNAs, which are involved in the regulation of fibroblast proliferation, apoptosis, and metabolism, therefore inhibiting fibrosis.^9^^,^^10^ For instance, RhoE itself is the promoter of fibroblast apoptosis and was confirmed to be a direct target of miR-200b. MMC could significantly downregulate miR-200b expression to induce apoptosis in human fibroblasts, thereby inhibiting scar formation.^7^ In the clinic, MMC was commonly applied by filling the filter channel with a cotton pad containing a certain concentration of MMC solution for a period of time. However, the instability of the drug concentration and complications including wound leakage, low IOP, choroidal detachment, corneal ulcers, intraocular inflammation, etc., greatly restricted the widespread application of this method.^11^^,^^12^ In this regard, developing advanced drug delivery platforms with stable and sustained MMC release capability for inhibiting postoperative fibrosis in GFS is in urgent need.

Considering the high water-solubility, high diffusivity, and low thermal stability of MMC, diverse organic/inorganic carriers have been developed for loading and in situ delivery of MMC.^13^^,^^14^ However, how to find a trade-off between prolonged release period and desired biodegradability, how to maintain the long-term bioactivity of MMC, and how to relieve toxic and immunological reaction still need to be taken into careful consideration. For instance, hydrogels including chitosan, albumin, and polybutylacrylate could maintain the in vivo bioactivity of MMC but could not release MMC for a relatively long time due to their fast water swelling/degradation property.^15^^–^^17^ Owing to the weak structural stability, lipid-based drug delivery systems are confronted with the same problem.^18^ Moreover, its drug loading amount is limited. Poly-2-hydroxyethylmethacrylate (PHEMA) hydrogel could release MMC in a sustained manner for over 10 days. However, the non-biodegradable property of PHEMA hydrogel greatly increased the risk of a second surgery.^19^ Extended MMC release could also be realized by utilizing mesoporous silica nanoparticles, but they may cause severe toxic and immunological reaction.^20^

Recently, electrospun nanofiber membranes (ENMs) have been frequently utilized for in situ drug delivery and have found applications in diverse fields, including tissue engineering, disease treatment, etc.^21^^,^^22^ Owing to the merits, including nanoscale size, porous structure, high specific surface area, and controlled payload, ENM-based drug delivery platform exhibits sustained/stable in situ drug delivery capability.^23^ In addition, some drugs are sensitive with temperature, light, air, etc., ENMs could, to some extent, prevent these drugs from losing their bioactivity. Polylactic acid (PLLA), a biocompatible and biodegradable polymer, has been processed into diverse materials for different biomedical applications. Especially, PLLA has been widely applied for eye-related disease treatment.^24^ For instance, Zhang et al. developed a PLLA-based amniotic fornical ring for ocular surface reconstruction.^25^ Levofloxacin loaded PLLA nanofiber membranes were utilized as a conjunctival alternative by Yan et al.^26^ In addition, owing to the variable molecular weight, helical structure, and degree of esterification, PLLA has been recognized as an excellent crude material for electrostatic spinning.^27^^,^^28^ However, the intrinsic hydrophobicity of PLLA, to some extent, affects cell adhesion on the surface of PLLA fiber and prolongs the degradation period of PLLA in vivo*.*^29^ In our previous work, we reported an Fmoc modified arginine-glycine-aspartic acid (RGD) peptide hydrogel for local drug delivery in rabbit eyes.^30^ The abundant hydrophilic side groups of the peptide endow it with superior water swelling capability whereas the RGD moiety facilitates cell adhesion.^31^

**Figure 1.**
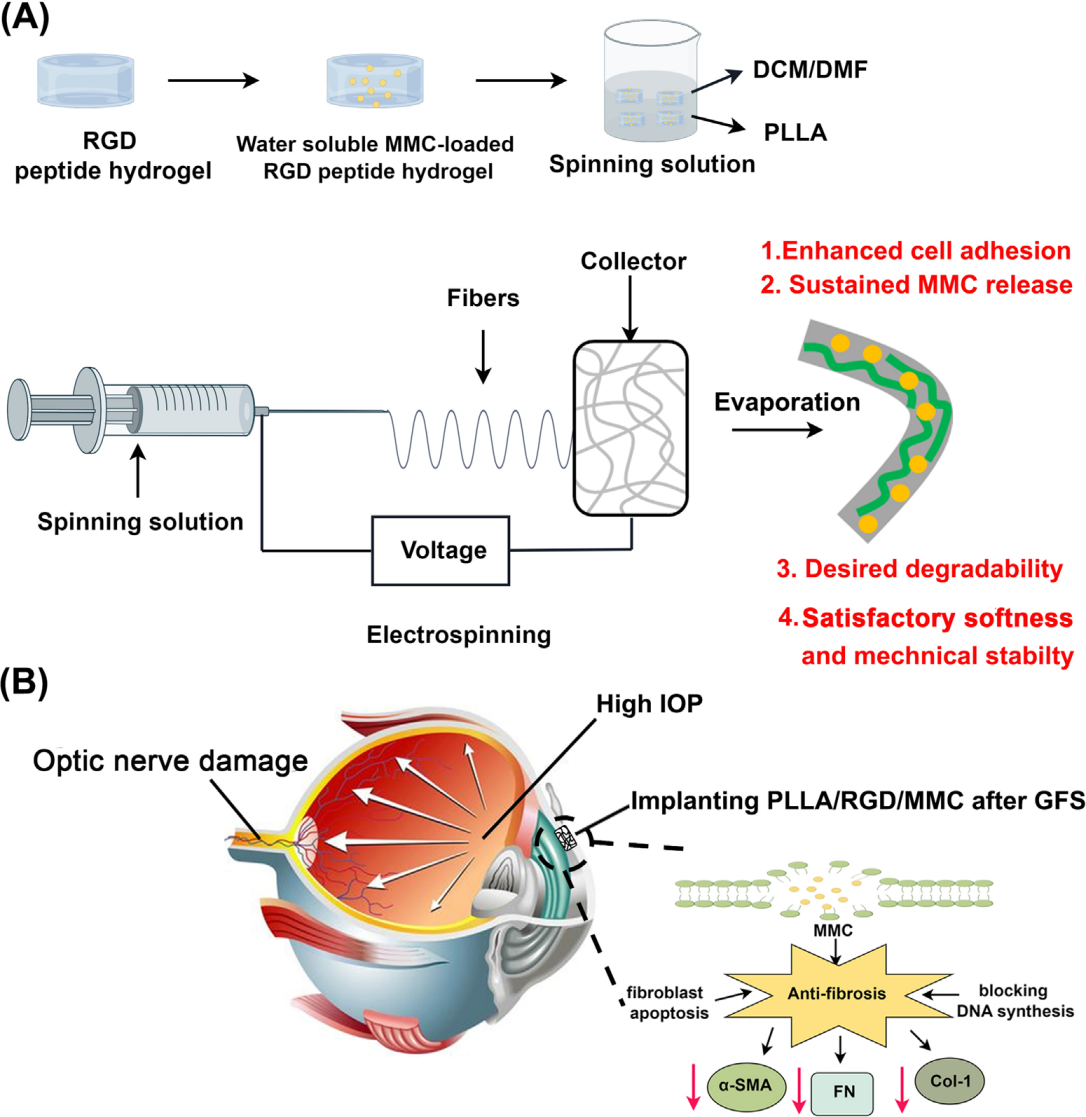
Figure by Figdraw. (**A**) Schematic diagram of the preparation process of the PLLA/RGD/MMC membrane and its internal structural composition; (**B**) the rationale behind the PLLA/RGD/MMC membrane suppressing scarring formation after GFS.

**Figure 2.**
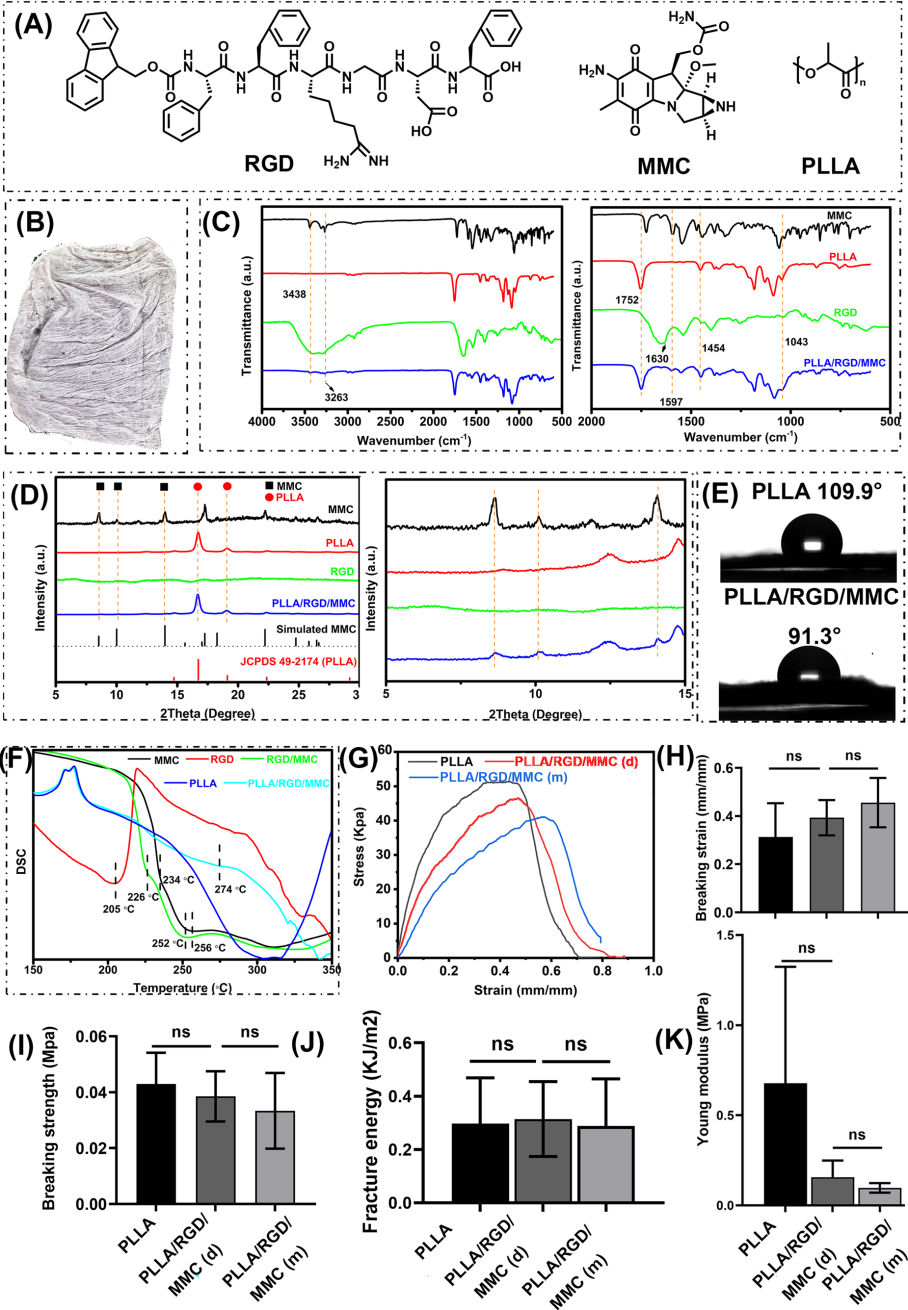
(**A**) Molecular structure of RGD, MMC, and PLLA; (**B**) digital photograph of as-prepared PLLA/RGD/MMC membrane; (**C**) FT-IR spectra; and (**D**) XRD profiles of MMC, PLLA, RGD, and the PLLA/RGD/MMC membrane; (**E**) water contact angle tests of PLLA membrane and the PLLA/RGD/MMC membrane; (**F**) DSC curves of MMC, RGD, RGD/MMC mixture, PLLA, and the PLLA/RGD/MMC membrane; (**G**) tensile stress-strain curves of PLLA membrane, dry PLLA/RGD/MMC membrane; (**D**) and moist PLLA/RGD/MMC membrane (m); Histograms of (**H**) fracture strain, (**I**) fracture stress, (**J**) fracture energy, and (**K**) Young's modulus obtained from (**G**).

In this regard, MMC-loaded PLLA/RGD membrane (termed PLLA/RGD/MMC) was fabricated through a simple wet spinning method. Here, DMF and DCM were utilized as solvent which could completely dissolve PLLA. Because direct exposure of MMC to DMF/DCM may undermine its bioactivity, MMC was first loaded in the RDG-peptide hydrogel and then the resultant hydrogel was homogeneously distributed in the DMF/DCM solution for subsequent electrostatic spinning and solvent evaporation (Fig. 1A). In this work, the composition, structure, water contact angle, biodegradability, MMC release behavior, and thermal stability of MMC was investigated in detail. In vitro cell culture assay was applied to investigate the cytocompatibility and cytotoxicity of the as-prepared material. A rabbit GFS model was utilized to investigate the capability of the as-prepared PLLA membrane in inhibiting postoperative fibrosis. Figure 2B demonstrates the potential mechanism of the PLLA/RGD/MMC membrane inhibiting scarring formation after GFS. We believed that the as-prepared MMC-loaded PLLA membrane could serve as an ideal biomaterial for inhibiting postoperative fibrosis in GFS in the future.

## Experimental Section

### Materials

The N-fluorenyl-9-methoxycarbonyl phenylalanine-phenylalanine-glycine-glycine-arginine-glycine-aspartic acid (Fmoc-FFGGRGD) short chain polypeptide powder (purity > 95%) was synthesized by Bioyeargene Biotechnology Ltd (Wuhan, China). PLLA (Mw ¼ 100 kilodalton [kDa], Mw/Mn ¼ 2.16) was obtained from Jinan Daigang Co., Ltd. (Jinan, China). MMC was purchased from Energy Chemical Co., Ltd. (Shanghai, China). The Masson’s Trichrome Staining kit was obtained from Google Biotech Co., Ltd. (Wuhan, China). The α-SMA, FN, and Col-1 were purchased from Sevier Biotechnology Co., Ltd. (Wuhan, China). All other chemical reagents, unless otherwise stated, were purchased from Sinopharm Regents Company (Wuhan, China).

### Preparation of the PLLA/RGD/MMC Membrane and the PLLA/RGD/MMC2 Membrane

Preparation of the PLLA/RGD/MMC membrane: 0.5*g* RGD powder was completely dissolved in 49.5 mL deionized water (37°C, 600 r/minute) to prepare 1.0 wt % RGD peptide hydrogel. Then, 10 mg MMC was completely dissolved in 1.0 wt % RGD peptide hydrogel and stirred for 30 minutes (room temperature, 120 r/minute). The PLLA solution was obtained by completely dissolving 0.5*g* PLLA in dichloromethane (DCM)/N, N dimethylformamide (DMF) mixture (v/v = 1.51 mL/1.06 mL) through agitation for 30 minutes (37°C, 120 r/minute). Then, the MMC/RGD peptide hydrogel solution was dripped into the DCM/DMF solution of PLLA to obtain PLLA/RGD/MMC electrospinning solution (room temperature, 120 r/minute). A 0.5-mm diameter needle was fitted to a 5.0 mL syringe and a syringe pump. The as-obtained mixture was drawn into the syringe to prepare PLLA/RGD/MMC membranes. A voltage of 16 to 20 kilovolt (kV) with a distance of 20 cm between the needle tip and the collector was applied. The flow rate of the polymer solution was controlled at 0.08 mm/minute. Afterward, the nanofiber membranes were placed in a vacuum oven for 24 hours to remove DCM, DMF, and water before proceeding to the next step of the experiment.

Preparation of the PLLA/RGD/MMC2 membrane: the preparation procedure of the PLLA/RGD/MMC2 membrane was the same as that of PLLA/RGD/MMC membrane but the content of MMC was increased to 40 mg.

Preparation of the PLLA/RGD membrane: the preparation procedure of the PLLA/RGD/MMC2 membrane was the same as that of PLLA/RGD/MMC membrane but the content of MMC was kept at 0.

### Physical/Chemical Characterizations

#### Scanning Electron Microscope 

The morphology of PLLA/RGD/MMC nanofiber membrane was observed by a scanning electron microscopy (SEM; JSM 7500F, Japan). The nanofiber membrane was first sputtered with gold for 120 seconds and then observed*.* The acceleration voltage was kept at 5 kV and the working distance was 7.5 mm. After obtaining the SEM images, the diameter of the nanofibers was analyzed by Image J software.

#### Transmission Electron Microscope and Energy Dispersive Spectrometer 

The milled PLLA/RGD/MMC nanofiber membrane powder was dispersed in anhydrous ethanol and subjected to ultrasonic treatment for 5 minutes. Then, the internal structure was observed by a field emission transmission electron microscopy (TEM; JEM-F200, JEOL, Japan). An energy dispersive spectrometer (EDS) was utilized to detect the elements of the fiber and EDS mapping was utilized to investigate the element distribution of the fiber.

#### X-Ray Diffraction 

The crystalline phases of the nanofiber membrane was studied by X-ray diffraction (XRD; SmartLab, Japan). First, the nanofiber membrane was ground into powder, and then the XRD test was carried out. In addition, we also tested PLLA, RGD, and MMC powders. The working conditions of XRD are CuK_0_ radiation through a rotating anode of 40 kV and 40 mA. The working condition of XRD was CuK_0_ radiation via a rotation anode at 40 kV and 40 milliampere (mA). The data were collected in a step of 0.1 degrees and a range of diffraction angles (2θ) from 5 degrees to 90 degrees.

#### Fourier Transform Infrared Spectrum 

The composition of PLLA/RGD/MMC nanofiber membranes and the interaction between different functional groups were studied by Fourier transform infrared spectrum (FT-IR; Fourier Transform, USA)*.* First, the PLLA/RGD/MMC nanofiber membrane was ground into powder, and then FT-IR testing was carried out using the potassium bromide method. At the same time, the FT-IR spectra of PLLA, RGD, and MMC were also detected.

#### Thermogravimetric Analysis and Differential Scanning Calorimetry 

Thermogravimetric analysis differential scanning calorimetry (TG-DSC; Diamond TG/DTA, Perkin Elmer, USA) was used to study the thermal behaviors. Briefly, the PLLA/RGD/MMC nanofiber membrane was ground into powder and then subjected to a TG-DSC test. We also plotted the TG-DSC curves of PLLA, RGD, MMC, and RGD/MMC mixture. In the air environment, the samples were heated at 10°C/minute in the temperature range of approximately 20°C to 800°C.

#### Water Contact Angle Test

The hydrophilicity of the nanofiber membranes were detected by an automatic contact angle tester (ThetaLite, Finland, Biolin).

#### Mechanical Properties Test

All the tests were conducted in air (25°C, 45% humidity) using a Universal Testing Machine (QingJi, Shanghai, China). All the force–displacement profiles were converted into nominal stress–strain curves.

Tensile Test: These samples were kept in a cuboid-shape (40 mm × 10 mm × 0.45 mm). The characteristic length (l_0_) was controlled at 30 mm. The stretch rate was maintained at 50 mm/minute. The stress (σ) was obtained by dividing the force (F) by the cross-sectional area, and the strain (ε) was obtained by dividing the stroke (l_t)_ by l_0_. The Young's modulus (E) was calculated according to the slope of the initial part (strain < 10%) of the tensile stress–strain curves. After each test, the machine directly gives the fracture stress and fracture strain. The fracture energy (W) was calculated by multiplying the area below the stress-strain curve of a sample by its characteristic length l_0_.

$$\begin{array}{c} \sigma =\frac{F}{w\times t} \end{array}$$

$$\begin{array}{c} ɛ=\frac{1_{t}}{1_{0}} \end{array}$$

$$\begin{array}{c} E=\frac{\sigma_{A}-\sigma_{B}}{{ɛ}_{A}-{ɛ}_{B}} \end{array}$$

$$\begin{array}{c} W=1_{0}\int_{0}^{{ɛ}_{f}}\sigma d_{ɛ} \end{array}$$

Fatigue-Resistance Test: The nominally stretched sample was stretched at a preset 3 mm and then unloaded. No resting time was applied between two successive loading-unloading tests. The operations were repeatedly conducted for 10 times. The toughness retention rate is calculated by dividing the dissipated energy after each tension by the ratio of the first loading and unloading test. The tensile rate of loading–unloading tests was maintained at 20 mm/minute. Each value was the mean for three replicates.

#### In Vitro Degradation Property

The prepared PLLA/RGD/MMC nanofiber membranes were immersed in a phosphate-buffered saline (PBS) with different pH values (pH 5.5, pH 7.4, and pH 9.3) at a temperature setting of 37°C, with a shaking speed (120 r/minute) for 4 weeks. At specific time points (i.e. 1, 3, 5, 7, 9, 11, 13, 15, 17, 19, 21, 23, 25, 27, and 29 days), samples were taken out, and after removing surface moisture with filter paper, they were weighed and subsequently returned to the PBS solution. The same operation was carried out at each sampling time point. The degradation rate was calculated using the following equations:

$$\begin{array}{c} \mathrm{Degradation}\;\mathrm{ratio}=\frac{M_{0}-M_{t}}{M_{0}} \end{array}$$

Here, M_0_ and M_t_ represent the initial weight and the weight of the sample at time point t, respectively. The results were averaged from three replicates.

#### In Vitro MMC Release Behavior

MMC powders were dissolved in PBS solution and then diluted to standard gradient concentrations of 5, 10, 15, 20, 25, 30, 35, 40, 45, 50, 55, 60, 65, 70, 75, 80, 85,90, 95, and 100 µg/mL. The absorbance was measured using a UV spectrophotometer (Lambda25) at a monochromatic wavelength of 364 nm. After zero adjustment, using blank PBS as a reference sample, we measured the absorbance of the standard MMC solutions. Measurements were repeated three times and averaged. The colorimetric plate was washed three times with PBS before changing to a different sample. We then plotted a scatter graph of MMC concentration against absorbance. The concentration range consistent with the Lambert-Beer law was determined. Within this range, the MMC solution with a smaller concentration gradient was configured again, and the final MMC absorbance standard curve was plotted (Supplementary Fig. S4).

Then, 50 mg of PLLA/RGD/MMC or PLLA/RGD/MMC2 nanofiber membrane was immersed in 20 mL PBS (pH 7.4). The suspensions were placed in 50 mL centrifuge tubes, and incubated in a constant temperature shaker at 37°C with a speed of 100 cycles/minute. Then, 4.0 mL of buffer was taken as a sample from the centrifuge tube, and 4.0 mL of blank PBS was added to maintain the total volume at different time points. The absorbance of the sample collected at different time points was measured by ultraviolet-visible spectrophotometry at 364 nm. Measurement was stopped when absorbance showed no change after three consecutive readings. The concentration of MMC in the buffer was calculated from the absorbance standard curve and the proportion of cumulative MMC released was calculated. We plotted drug release curves, repeated measurements three times with OriginPro2021 software, and fitted them with mathematical models.

### In Vitro Cell Culture Assay

Human embryonic Tenon’s capsule fibroblasts (HFTFs) were purchased from the cell bank of the Shanghai Academy of Sciences, China. HFTFs were incubated in DMEM supplemented with 10% fetal bovine serum (FBS; Israel Biological Industries Company), 100 U/mL penicillin and 100 U/mL streptomycin in a 37°C, 5% CO2 cell incubator. The culture medium was changed every 3 days.

CCK-8 test: The PLLA/RGD membrane, the PLLA/RGD/MMC membrane, and the PLLA/RGD/MMC2 nanofiber membrane were cut into circular samples with a diameter of 5 mm, and both sides of these samples were disinfected under UV light for 4 hours. They were then immersed in 5 mL of DMEM for 48 hours. After that, the DMEM was collected. Then, 100 uL of cell suspension was added to a 96-well plate, evenly spread, and incubated at 37°C for 24 hours. When HFTFs were pooled and merged to 70%, 10 uL of the collected DMEM was added to each well and fully mixed. After 24 hours, 48 hours, and 72 hours of incubation, 10 uL of CCK8 solution was added to each well in subdued light, followed by incubation for 1 hour at 37°C. The absorbance was then measured at 450 nm using a microplate reader.

Live/dead cell staining: The PLLA/RGD membrane, the PLLA/RGD/MMC membrane, and the PLLA/RGD/MMC2 membrane were cut into circular samples with a diameter of 5 mm, and both sides of these samples were disinfected under UV light for 4 hours. After washing with DMEM, the cells were placed into 24-well plates and 500 µL of cell suspension was added to each well. Detection with the prepared live/dead stain kit (Beyotime Biotechnology, Shanghai, China) was performed on the third and fifth days. Three replicate experiments were performed in each group.

### In Vivo Animal Assay

All animal procedures followed the guidelines outlined in the Association for Research in Vision and Ophthalmology (ARVO) Policy on the use of animals in ophthalmology and vision research. This study was approved by the Institutional Animal Care Committee of the China Three Gorges University (No.: NO.42817300002679). Twenty-four adult New Zealand white rabbits (weight range = 2.0–3.0 kg) were used for the experiments. All animals had no conjunctival congestion and corneal opacity. The rabbits were adapted for 1 week before experimentation. Based on the drug release curve, the nanofiber membrane (PLLA/RGD/MMC2) loaded with 4% MMC was selected for the next in vivo animal experiment. Then, 24 New Zealand white rabbits were randomly divided into four groups: the sham group, the PLLA/RGD nanofiber membrane group, the 0.4 mg/mL MMC cotton pad group, and PLLA/RGD/MMC2 nanofiber membrane group.

Establishment of the Rabbit Glaucoma Filtration Surgery Model: The animals were anesthetized with an intravenous injection of pentobarbital sodium (30 mg/kg; Solarbio, Beijing, China). Procaine hydrochloride was dripped for ocular surface anesthesia, and conjunctival flap based on limbus was made to separate subconjunctival fascia and other tissues to fully expose the sclera. After adequate cauterization of the scleral surface for hemostasis, a 4 mm × 4 mm square scleral flap of approximately ½ the scleral thickness was created based at the corneal limbus. The flap was dissected anteriorly until the clear cornea was reached, followed by an additional 1 mm dissection into the transparent cornea. Anterior chamber puncture was performed in the temporal transparent cornea, and aqueous humor was slowly released to reduce IOP. Approximately 1.5 mm × 2 mm of limbal tissue containing the trabecular meshwork was excised, and a peripheral iridectomy was performed. The PLLA/RGD membrane or PLLA/RGD/MMC2 membrane was trimmed to 3 mm × 3 mm and placed under the scleral flap. Both ends of the scleral flap were secured to the sclera with one suture each using 10-0 nylon sutures (Alcon, USA). The Tenon’s capsule was closed with interrupted 10-0 nylon sutures, and the conjunctiva was continuously sutured with 8-0 absorbable suture (Ethicon, Inc., USA).The sham group underwent the same surgical procedure, except that the scleral flap was secured directly without implanting any membrane. In the MMC cotton pad group, 0.4 mg/mL MMC cotton sheets were placed under the scleral flap for 2 minutes, and then removed, and washed with a large amount of normal saline. To reduce postoperative inflammation of the surgical eye, an appropriate amount of Tobramycin Dexamethasone Eye Ointment (once a day) and Levofloxacin eye drops (4 times a day) were used on the surface of surgery for the first week. Within each group, a rabbit was randomly selected as the observation target, and photographs were taken on days 7, 14, and 28 to observe the filtration bubble bulge status and healing.

Histology and immunohistochemistry: New Zealand white rabbits were euthanized 28 days after the establishment of the rabbit eye surgical model. Animals were euthanized at post operative day (POD) 28 via intravenous injection of 150 mg/kg pentobarbital sodium (administered through the marginal ear vein) to ensure rapid unconsciousness (<30 seconds) followed by cardiac arrest. This method complies with the AVMA Guidelines for Euthanasia (2020). Ocular tissues were collected, rinsed with PBS, fixed in 4% paraformaldehyde, dehydrated with graded ethanol, and embed in paraffin. After paraffin slices’ preparation, the slices were stained with Masson Trichrome (Google Biotech, Wuhan, China). The images were taken under a microscopy (Panoramic MIDI, three-dimensional [3D] HISTWCH). The site of damage was determined by Masson staining. We took sections from the same locations for immunohistochemical staining. Paraffin sections were deparaffinized with xylene and rehydrated in gradient ethanol. Sections were washed with distilled water and then immersed in 0.01 mol / L sodium citrate buffer (pH 6.0) for epitope repair. Sections were blocked with 3% BSA and stained after incubation with the primary antibody (alphasmooth muscle actin [α-SMA] at 1:1000 dilution, collagen I [Col-1] at 1:500 dilution and fibronectin [FN] at 1:200 dilution; Servicebio, Wuhan, China). The average optical density of α-SMA, FN, and Col-1 was evaluated by ImageJ software to evaluate their expression level.

### Statistical Analysis

Experimental results were analyzed using the statistical software Graphpad Prism 9.0. All data were presented as mean ± standard deviation (SD). If the experimental results followed the normal distribution, 1-way analysis of variance (ANOVA) was used for comparing multiple groups. If the experimental results did not follow the normal distribution, the Kruskal-Wallis test was used to compare differences between groups. *P* < 0.05 was considered statistically significant.

## Results and Discussions

### Physical/Chemical Properties of As-Prepared PLLA/RGD/MMC Membrane

Figure 2A demonstrates the molecular structure of the three basic components of the PLLA/RGD/MMC membrane, namely, RGD, MMC, and PLLA. Figure 2B shows the digital photograph of as-prepared electrospinning membrane. From the FT-IR data (Fig. 2C), it could be found that MMC had 3 typical absorbance peaks at 3438 cm^−1^, 3263 cm^−1^, and 1597 cm^−1^, which were attributed to the NH_2_ group on the hexatomic ring, NH_2_ group of the primary amide on the carbamoyl structure, and the C = C backbone of the quinone ring in MMC, respectively.^32^^,^^33^ PLLA also exhibited 3 typical absorbance peaks at 1752 cm^−1^, 1454 cm^−1^, and 1043 cm^−1^, which were assigned to –C = O, –CH_3_, and –OH groups of PLLA, respectively.^34^ Obviously, the above-mentioned peaks could also be found in the FT-IR spectrum of the PLLA/RGD/MMC membrane, proving that MMC has been successfully incorporated into the membrane. In order to figure out whether RGD has been incorporated into the membrane, we compared the peak intensity of I_1043_/I_1752_ of PLLA and the PLLA/RGD/MMC membrane and found that the ratio was decreased from 82.43/74.56 to 71.17/72.12, indicating that RGD peptides were included in the PLLA/RGD/MMC membrane. It could also be found that the peak at 1630 cm^−1^ (C = N) in the FR-IR spectrum of RGD disappeared in that of the PLLA/RGD/MMC membrane, which might be due to the hydrogen bond interaction between RGD and MMC.^35^ In addition, the absorbance peaks of DMF and DCM could not be observed, indicating that the two toxic reagents have been completely removed in the evaporation process. From the XRD profiles (Fig. 2D), it could also be concluded that MMC crystallites were distributed within the PLLA/RGD fiber.^32^

Owing to the incorporation of RGD, the water contact angle of the PLLA/RGD/MMC membrane, compared to pure PLLA membrane, was decreased from 109.9 degrees to 91.3 degrees (Fig. 2E), demonstrating enhanced hydrophilicity of the membrane. In Supplementary Figure S1 (supporting information), the MMC mass percentage of the PLLA/RGD/MMC membrane was calculated from the TG curves (Supplementary Fig. S1) and the final value was determined to be 0.97%, which was approximated to our initial feeding composition (1%). It indicated that the encapsulation efficiency of MMC within the PLLA/RGD/MMC membrane was near 100%. In Figure 2F, the thermal behaviors of the PLLA/RGD/MMC were investigated in detail. Pure MMC exhibited 2 exothermic peaks at approximately 234°C and 256°C; RGD powder showed 1 exothermic peak at 205°C. When MMC was dispersed in the RGD hydrogel, the resultant RGD/MMC mixture showed 2 exothermic peaks at 226°C and 252°C. In contrast, when MMC was hybridized with RGD and PLLA, the first exothermic peak was moved to 274°C. Obviously, RGD molecules within the PLLA/RGD/MMC membrane could not enhance the thermal stability of MMC but PLLA, to some extent, prevented the MMC from losing its bioactivity under heat. In the clinic, mechanical properties of the PLLA/RGD/MMC membrane are of vital importance for its practical use. Therefore, we investigated its tensile behaviors and analyzed corresponding data in detail (Figs. 2H–K). The incorporation of RGD within the PLLA fiber did not jeopardize its strength and toughness, its fracture strength and fracture energy were still as high as 0.37 MPa and 0.35 kJ/m^2^, respectively (see Figs. 2I, 2J). However, the fracture strain of the PLLA/RGD/MMC membrane, compared to the pure PLLA membrane, was increased by 20% whereas the Young's modulus was decreased by 55% (see Figs. 2H, 2K). Obviously, RGD improved the softness of the PLLA/RGD/MMC membrane, beneficial for its clinical applications. Moreover, when the PLLA/RGD/MMC membrane was exposed to moisture, its mechanical behaviors did not change extensively, indicating the potential high mechanical/structural stability of the PLLA/RGD/MMC membrane in vivo. Supplementary Figure S2 shows the anti-fatigue capability of the moist PLLA/RGD/MMC membrane. Obviously, the PLLA/RGD/MMC membrane could maintain the initial strength under successive mechanical deformation (strain of 0.1, no resting time) but was prone to lose its energy dissipation capability in repeated tension.

**Figure 3.**
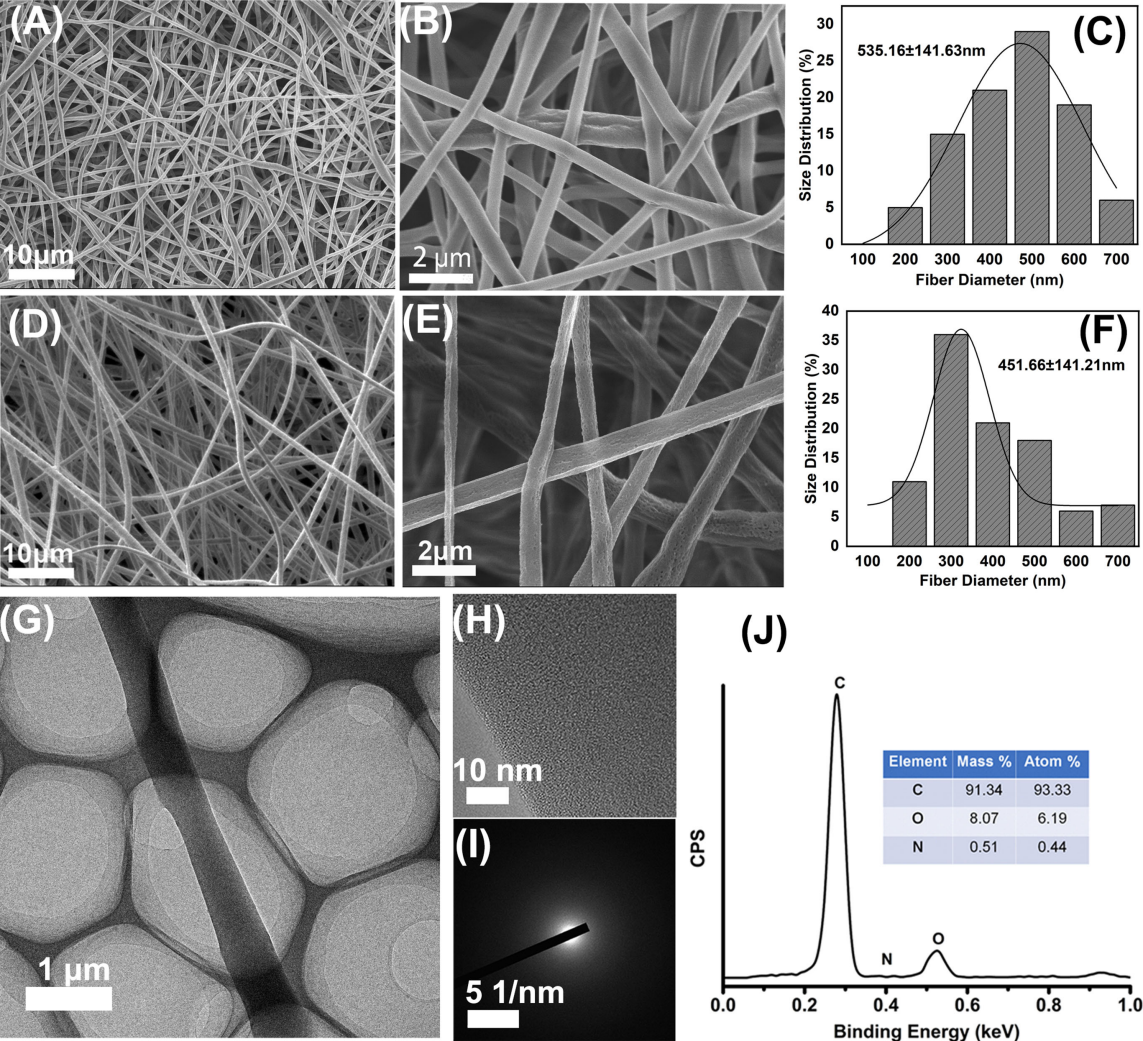
SEM images of (**A,** **B**) pure PLLA fiber and (**D,** **E**) the PLLA/RGD/MMC fiber, (**C**) and (**F**) show the diameter distribution of pure PLLA fiber and the PLLA/RGD/MMC fiber obtained from **A** and **D**), respectively; (**G**) TEM image of the PLLA/RGD/MMC fiber, (**H**) and (**I**) show the high-resolution TEM image and the selected electron diffraction (SAED) image; (**J**) EDS curve of the PLLA/RGD/MMC fiber. The insert exhibits the mass percentage and atom percentage of C, O, and N.

In Figures 3A–F, the microscopic morphology of pure PLLA membrane and the PLLA/RGD/MMC membrane was demonstrated in detail using an SEM. Both of the two membranes were porous, which were favorable for cell adhesion and transport of oxygen and nutrition. The incorporation of RGD seemed to decrease the dimensional homogeneity of the fibers but increase the porosity of the membrane. Compared to the pure PLLA fiber, the average diameter of the PLLA/RGD/MMC fiber was decreased from 535 nm to 451 nm. The finer fiber diameter and higher porosity of the PLLA/RGD/MMC membrane, in principle, also facilitate cell adhesion. In addition, we also observed the internal structure of the PLLA/RGD/MMC fiber through a TEM (Fig. 3G). Although the MMC crystallites were detected in the XRD profile of the PLLA/RGD/MMC membrane (see Fig. 2D), no obvious aggregates were observed in the TEM image of the PLLA/RGD/MMC fiber (see Fig. 2G), proving that RGD and MMC were homogeneously distributed within the PLLA matrix. The EDS analysis (Fig. 3H) and EDS mapping (Supplementary Fig. S3) also proved the uniform distribution of RGD and MMC within the PLLA fiber.

### Degradation Behaviors and MMC Release Behaviors of the PLLA/RGD/MMC Membrane

**Figure 4.**
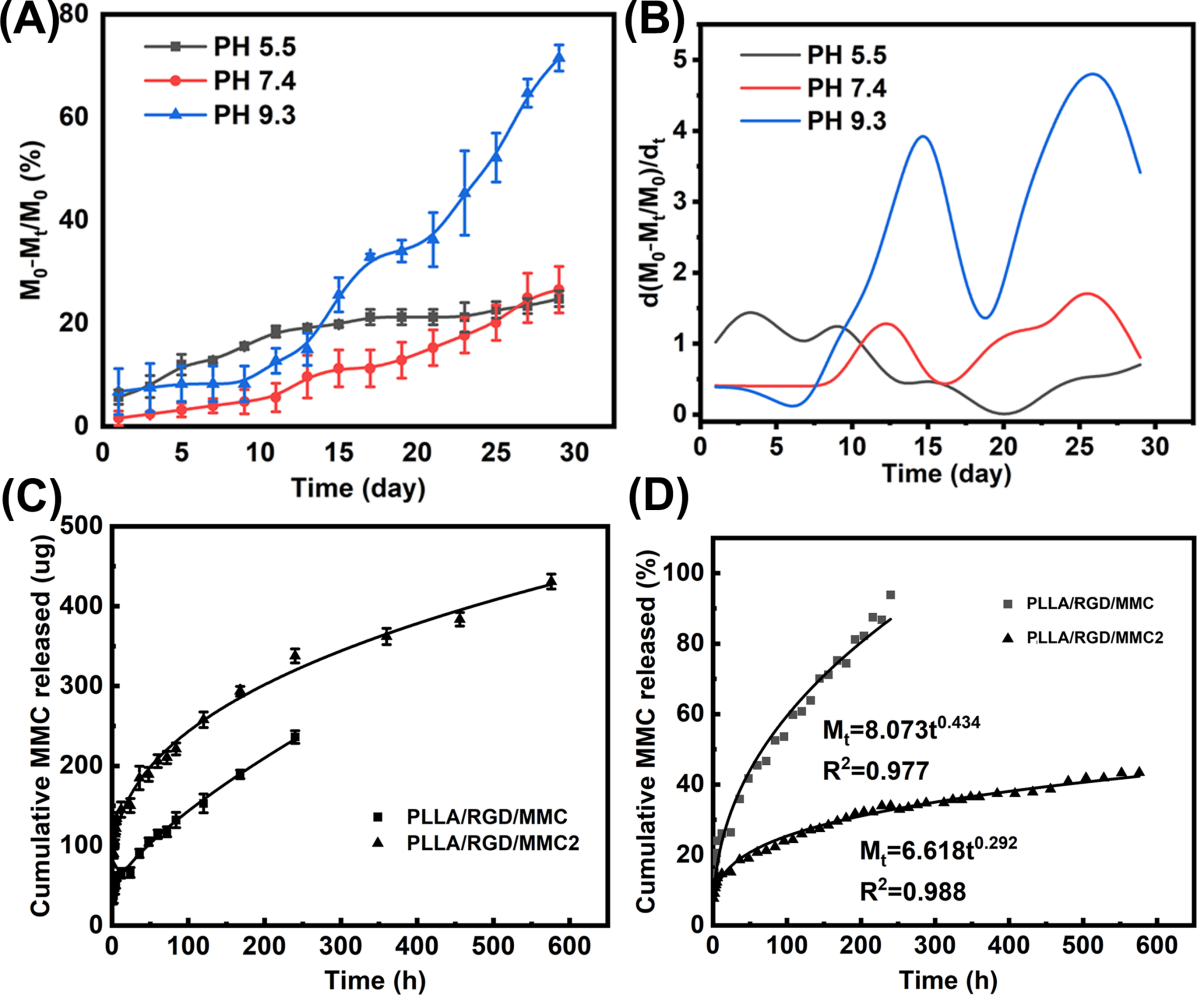
(**A**) In vitro degradation curves of the PLLA/RGD/MMC membrane in PBS with pH of 5.5, 7.4, and 9.3. (**B**) Shows the corresponding degradation rate-time curves; (**C**) MMC cumulative release curves of the PLLA/RGD/MMC and PLLA/RGD/MMC2 membranes in simulated eye environment (pH 7.4, temperature 37°C). (**D**) Shows the fitted curves using a Korsmeyer-Peppas model.

As is known, in vivo degradation behaviors and MMC release behaviors of the PLLA/RGD/MMC membrane determine its clinical applications. Its degradation time, in principle, should fit well with the treatment period of GFS. From Figure 4A, it could be found that after 28 days’ of degradation near 20% of the membrane was degraded in a simulated eye environment (pH 7.4). Here, it could be concluded that the PLLA/RGD/MMC membrane could provide a stable and prolonged treatment time for inhibiting postoperative fibrosis in GFS. Additionally, we also investigated the degradation mechanism of the membrane. From Figure 4B, it could be found that the degradation rate of the membrane in PBS with pH of 9.3 or 5.5 was increased, which was because of the hydrolysis of ester bond in acidic or alkaline environments could be accelerated.^36^ Thus, it could be concluded that the degradation of the membrane was owing to the hydrolysis of PLLA fiber. In Figure 4C, the in vitro MMC release curve was plotted using a standard curve method (see Supplementary Fig. S4), from which we found that the as-prepared PLLA/RGD/MMC membrane could only release MMC for less than 300 hours. Obviously, the MMC release time is much less than the treatment time of GFS. Thus, we increased the initial feeding content of MMC from 1% to 4%, and prepared another membrane termed PLLA/RGD/MMC2. Supplementary Figure S5 exhibited the morphology, fiber diameter, and contact angle of the PLLA/RGD/MMC2 membrane, which were nearly the same with those of the PLLA/RGD/MMC membrane.

Through increasing the MMC content, the resultant PLLA/RGD/MMC2 membrane could release MMC in a sustained manner for over 600 hours. Thus, in the following animal assays, the PLLA/RGD/MMC2 membrane, instead of the PLLA/RGD/MMC membrane, was applied. To figure the MMC release mechanism, we, herein, utilized five models to fit the MMC release curves, namely, zero-order model (Supplementary Fig. S6), first-order model (Supplementary Fig. S7), Kopcha model (Supplementary Fig. S8), Higuchi model (Supplementary Fig. S9), and Korsmeyer-Peppas model (Fig. 4D). From these figures, it could be found that the MMC release curves fitted well with Higuchi model and Korsmeyer-Peppas model. In Supplementary Figure S2 (supporting information), the applications of different models were summarized in detail. Higuchi model is commonly utilized to describe the drug release from an insoluble matrix based on the Fickian diffusion, whereas the Korsmeyer-Peppas model is commonly used to describe drug release from the polymeric system. The difference between the Higuchi model and the Korsmeyer-Peppas model is that the latter takes polymer degradation into consideration. Thus, we finally applied the Korsmeyer-Peppas model to explain the MMC release mechanism of the PLLA/RGD/MMC and PLLA/RGD/MMC2 membranes. Table 1 demonstrates the related fitted parameters of the Higuchi model and the Korsmeyer-Peppas model. From the n value (<0.5), it could be concluded that MMC release from the membranes were owing to the Fickian diffusion instead of the synergy of MMC diffusion and PLLA erosion.

**Table 1.** Related Fitted Parameters of the Higuchi Model and the Korsmeyer-Peppas Model

		Korsmeyer-Peppas Model	
Sample	Higuchi Model R^2^	R^2^	n
PLLA/RGD/MMC	0.987	0.977	0.434
PLLA/RGD/MMC2	0.986	0.988	0.292

### Cytocompatibility of the PLLA/RGD/MMC and PLLA/RGD/MMC2 Membranes

**Figure 5.**
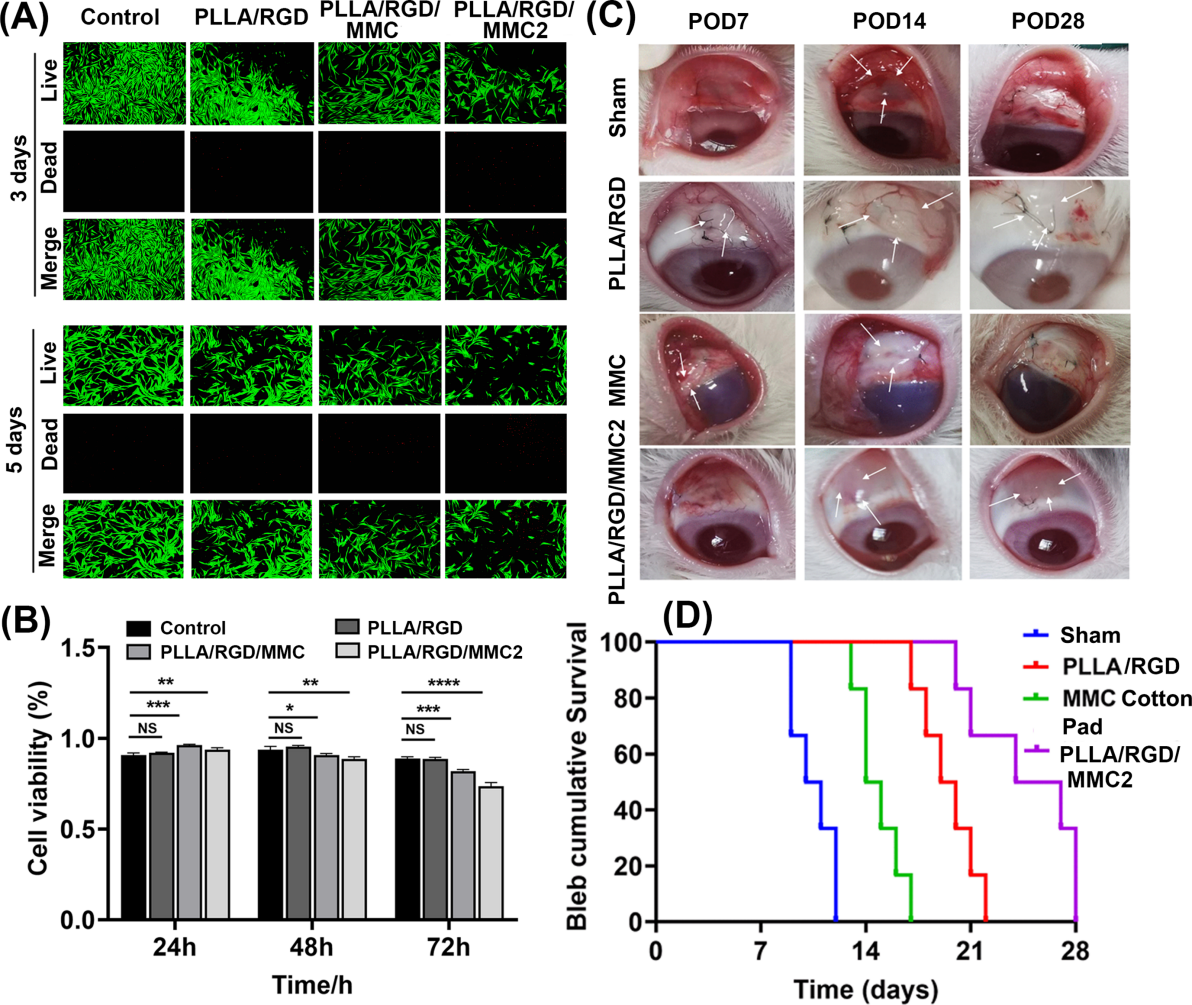
(**A**) Live/dead cell staining. (**B**) Histogram showing the results of the CCK-8 assay. *P < 0.05, **P < 0.01, ***P < 0.005, ****P < 0.001. (**C**) Representative images show the appearance of filtration blebs in sham group, the PLLA/RGD membrane group, the MMC cotton pad group, and the PLLA/RGD/MMC2 membrane group at POD 7, 14, and 28 after GFS. The *white arrows* in the pictures of the sham, PLLA/RGD, MMC cotton pad, and PLLA/RGD/MMC2 groups show the filtration blebs. (**D**) Kaplan-Meier survival curves for the four groups.

From the results of live/dead cell staining (Fig. 5A) and the cell viability test (Fig. 5B), it could be found that although DCM and DMF were used in the preparation process of the membranes, they were compatible and nontoxic with HFTFs. Compared with the PLLA/RGD/MMC group, the PLLA/RGD/MMC2 group demonstrated slightly decreased cell viability, but the cells still maintain high proliferation potential. Thus, the PLLA/RGD/MMC2 membrane was suitable for in vivo assay.

### Bleb Characteristics and Survival Time Evaluation

We assessed the impact of different treatments on the survival of filtration blebs following GFS. Figure 5C shows the appearance of filtration blebs of the sham group, the PLLA/RGD membrane group, the MMC cotton pad group, and the PLLA/RGD/MMC2 membrane group at day 7, day 14, and day 28 after GFS. Evaluation of the filtration bleb morphology by IBAGS was summarized in Table 2.^37^^–^^39^ Through analyzing and comparing the height, width, vascularization, steaming of the filtering bled (see Table 2), we found that the filtering bleb state of the PLLA/RGD/MMC2 membrane group was the best compared with the other three groups, indicating that the PLLA/RGD/MMC2 membrane could effectively maintain the long term unobstructed of the filtering channel and showed significant advantage over conventional MMC cotton pad. The same conclusion could be drawn through comparing the bleb survival time in Kaplan–Meier survival curves. Compared with the sham group, the PLLA/RGD membrane group, and the MMC cotton pad group, the PLLA/RGD/MMC2 group demonstrated a longer bleb survival time. The median survival time of the sham group, the PLLA/RGD group, the MMC cotton pad group, and the PLLA/RGD/MMC2 group were 10.5, 19.5, 14.5, and 25.5 days, respectively.

**Table 2.** Indiana Bleb Appearance Grading Scale in the Control Group, the PLLA/RGD Membrane Group, the MMC Cotton Pad Group, and the PLLA/RGD/MMC2 Group

Group	POD 7	POD 14	POD 28
Sham	H0, E1, V2, S0	H1, E1, V1, S0	H0, E0, V1, S0
PLLA/RGD	H1, E2, V2, S0	H2, E2, V2, S0	H2, E2, V1, S0
MMC	H1, E1, V2, S0	H1, E1, V2, S0	H0, E1, V1, S0
PLLA/RGD/MMC2	H2, E2, V3, S0	H2, E3, V3, S0	H2, E2, V2, S0

H, height, H0 (flat) to H4 (high); E, extent, E0 (less than 1 clock hour) to E3 (more than 4 clock hours); V, vascularity, V0 (avascular) to V4 (extensive vascularity); S, Siedel, S0 (no leak) to S2 (streaming).

### Histology and Immunohistochemistry

**Figure 6.**
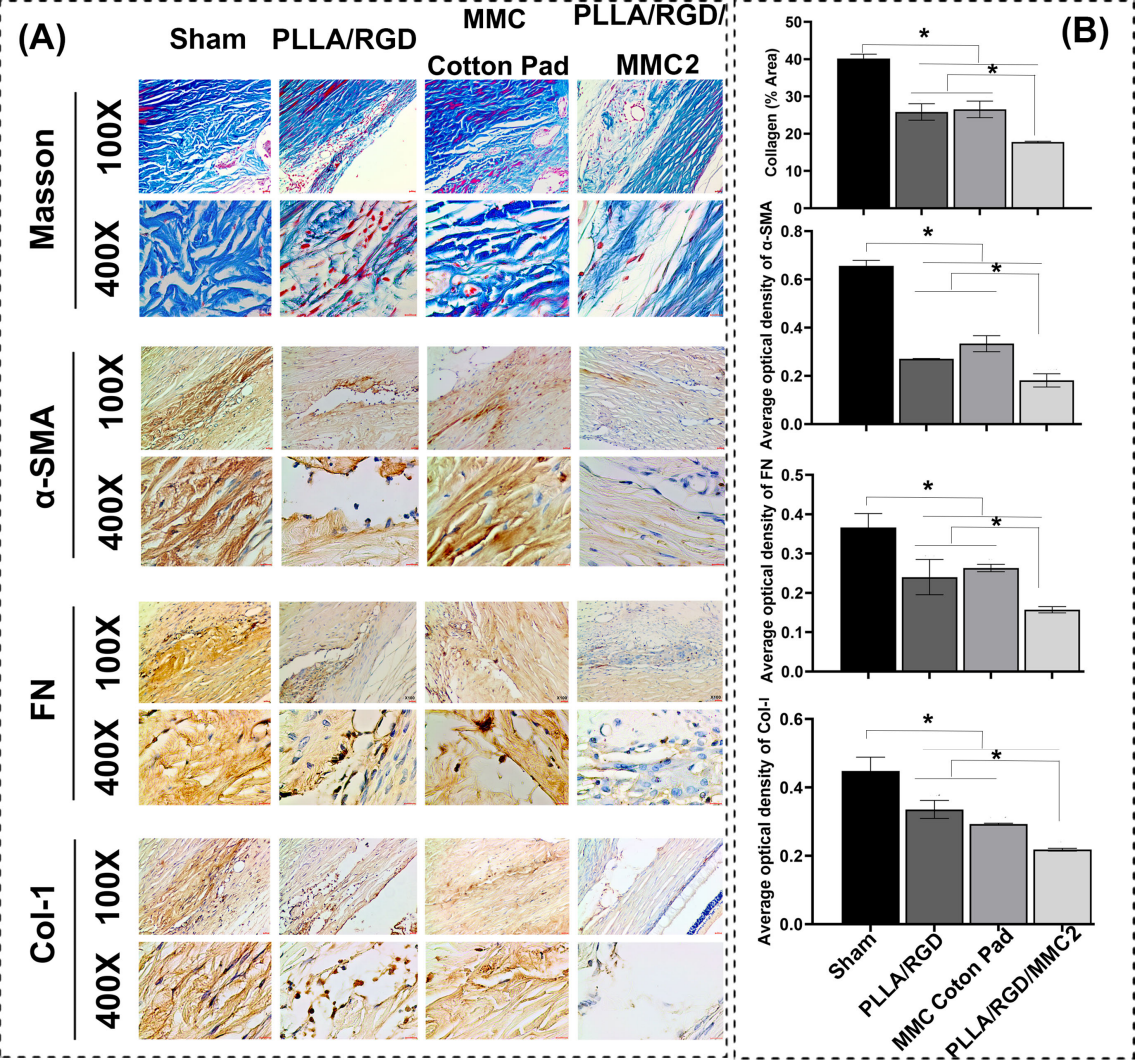
(**A**) Scar assessment of the sham group, the PLLA/RGD membrane group, the MMC cotton pad group, and the PLLA/RGD/MMC2 membrane group on day 28 after GFS through Masson staining and immunohistochemistry staining including α-SMA, FN, and Col-1. (**B**) quantitative analysis of **A**. *P < 0.05.

It is widely believed that scar formation following GFS is primarily associated with fibroblast and myofibroblast proliferation.^40^ Myofibroblast proliferation is characterized by the expression of α-SMA.^41^ Furthermore, in response to local tissue injury, myofibroblasts synthesize and secrete abundant extracellular matrix (ECM) proteins, including collagen types I, III, IV, and V, as well as fibronectin (FN), facilitating tissue remodeling.^42^ To investigate these components, Masson’s trichrome staining and immunohistochemistry for α-SMA, FN, and collagen I (Col-1) were performed at POD 28 (Fig. 6).

Masson’s trichrome staining results revealed dense and disorganized collagen fibers in the sham group, indicating marked scar formation after GFS. In contrast, collagen fibers in the other three treatment groups were more regularly arranged and sparser, demonstrating that both the PLLA/RGD membrane and the MMC cotton pad effectively inhibited scar formation to some extent. Quantitative analysis of the proportion of newly formed collagen fibers at the surgical site relative to the total conjunctiva-sclera section area showed that the PLLA/RGD/MMC2 membrane suppressed collagen fiber formation more effectively than the MMC cotton pad, highlighting its significant therapeutic potential. Subsequently, we compared the expression of α-SMA, FN, and Col-1 in scleral tunnel tissues from the four groups. Consistent with the Masson staining findings, the PLLA/RGD/MMC2 group exhibited a greater capacity to inhibit the expression of α-SMA, FN, and Col-1 compared with the other three groups.

In this research, the potential toxicity and safety risks associated with long-term exposure to MMC also need to be considered. To minimize the toxicity risk of MMC, we designed and considered the following aspects: (1) the concentration of MMC used in this study was carefully optimized through literature review and preliminary experiments, which is significantly lower than the concentrations reported in the literature to be associated with significant toxicity. We chose a low dose to balance its anti-proliferative effect and safety. According to the literature, the safe concentration range of MMC for ocular applications is 0.2 to 0.4 mg/mL.^43^^–^^46^ Our dosage selection was based on previous studies, which demonstrated that similarly low doses of MMC exhibited favorable safety profiles in animal models. Low concentrations of MMC are known to inhibit cell proliferation with low cytotoxicity.^47^^–^^48^ (2) Traditional drug delivery methods cause an initial sudden release of the drug within a short period. The PLLA/RGD/MMC drug delivery system we used was designed to provide controllable and slow drug release. The drug delivery system eliminates the risk of acute toxicity associated with sudden exposure to high drug concentrations. The drug sustained-release system designed by us can maintain the drug concentration above the therapeutic level and below the toxic level for a long time, so as to achieve the purpose of efficient and low-toxicity treatment. (3) During the operation, the nanofiber membrane patch is positioned and secured under the scleral flap, ensuring the drug-loaded system is accurately placed within the target tissue area. This approach minimizes drug diffusion to surrounding structures and systemic absorption. (4) As demonstrated in the results section of our paper, we conducted detailed histopathological analyses of the surgical site and surrounding critical tissues. The results showed that no significantly increased inflammatory responses or abnormal tissue necrosis were observed in our treatment group. None of the animals showed any clinical signs of systemic toxicity throughout the entire experimental period. These data indicate that no significant local or systemic toxicity was caused by the doses and delivery systems used.

## Conclusions

In this study, we developed a PLLA/RGD/MMC membrane using an electrospinning technology and applied it to maintain unobstructed filter channel and inhibit scaring formation after GFS. The results of FT-IR, XRD, SEM, TEM, EDS, etc., demonstrated that: (i) as-prepared membrane was highly porous and its porosity was higher than pure PLLA membrane; (ii) RGD and MMC were homogeneously dispersed in the PLLA matrix; and (iii) the incorporation of RGD increased the hydrophily of the PLLA fiber. The TG test indicated that the MMC encapsulating efficacy of the membrane was approximated to 100%, and the DSC curves demonstrated that the PLLA matrix could prevent MMC from losing its bioactivity under heat to some extent. The in vitro degradation assay indicated that the membrane could be slightly degraded in a simulated eye environment, and the rationale behind degradation was due to the hydrolysis of ester bond of PLLA. In vitro MMC release curves indicated that through adjusting the initial MMC feeding content the membrane could release MMC in a sustained manner for over 25 days. In addition, MMC release from the membrane followed a Korsmeyer-Peppas model, and was owing to Fickian diffusion instead of the synergy of MMC diffusion and PLLA erosion. Live/dead cell staining and CCK-8 assays showed the superior cytocompatibility of the membrane with HFTFs, whereas bleb characteristics and survival time evaluation proved that the membrane could maintain long term, unobstructed condition of the filter channel after GFS. Masson staining and immunohistochemistry staining of α-SMA, FN, and Col-1 show that the membrane, compared to the conventional MMC cotton pad, had stronger capability in preventing scarring formation after GFS.

Although this study indicated the potential of the PLLA/RGD/MMC2 membrane in anti-scarring applications, further research is needed to gain a deeper understanding of the specific mechanisms involved. We need further research to investigate the process of scar tissue formation and how MMC, PLLA, and RGD peptide hydrogels affect scar-related signaling pathways. The results of this study provide new insights into the treatment of scarring after GFS. In future research, we could consider further improvement of electrospun nanofiber materials to improve the efficiency and durability of drug release. Furthermore, other molecular and signaling pathways that may influence the anti-scar mechanisms could be investigated to gain insight into the biological basis of scar formation.

## Supplementary Material

Supplement 1

## References

[1] Stein JD, Khawaja AP, Weizer JS. Glaucoma in adults—screening, diagnosis, and management: a review. *JAMA*. 2021; 325(2): 164–174. 33433580 10.1001/jama.2020.21899

[2] Heijl A, Leske MC, Bengtsson B, et al. Reduction of intraocular pressure and glaucoma progression: results from the Early Manifest Glaucoma Trial. *Arch Ophthalmol*. 2002; 120(10): 1268–1279. 12365904 10.1001/archopht.120.10.1268

[3] Conlon R, Saheb H, Ahmed IIK. Glaucoma treatment trends: a review. *Can J Ophthalmol*. 2017; 52(1): 114–124. 28237137 10.1016/j.jcjo.2016.07.013

[4] Agnifili L, Sacchi M, Figus M, et al. Preparing the ocular surface for glaucoma filtration surgery: an unmet clinical need. *Acta Ophthalmol*. 2022; 100(7): 740–751. 35088941 10.1111/aos.15098

[5] van Mechelen RJ, Wolters JE, Herfs M, et al. Wound healing response after bleb-forming glaucoma surgery with a SIBS microshunt in rabbits. *Transl Vis Sci Technol*. 2022; 11(8): 29. 10.1167/tvst.11.8.29 PMC9428362 36018582

[6] Santhiago MR, Netto MV, Wilson SE. Mitomycin C: biological effects and use in refractive surgery. *Cornea*. 2012; 31(3): 311–321. 22157595 10.1097/ICO.0b013e31821e429d

[7] Sun Y, Ge Y, Fu Y, et al. Mitomycin C induces fibroblasts apoptosis and reduces epidural fibrosis by regulating miR-200b and its targeting of RhoE. *Eur J Pharmacol*. 2015; 765: 198–208. 26254780 10.1016/j.ejphar.2015.08.002

[8] Gray SD, Tritle N, Li W. The effect of mitomycin on extracellular matrix proteins in a rat wound model. *Laryngoscope*. 2003; 113(2): 237–242. 12567075 10.1097/00005537-200302000-00008

[9] Aprelikova O, Green JE. MicroRNA regulation in cancer-associated fibroblasts. *Cancer Immunol Immunother*. 2012; 61: 231–237. 22083346 10.1007/s00262-011-1139-7 PMC3704172

[10] Yang S, Banerjee S, de Freitas A, et al. Participation of miR-200 in pulmonary fibrosis. *Am J Pathol*. 2012; 180(2): 484–493. 22189082 10.1016/j.ajpath.2011.10.005 PMC3349843

[11] Lim R . The surgical management of glaucoma: a review. *Clin Exp Ophthalmol*. 2022; 50(2): 213–231. 35037376 10.1111/ceo.14028

[12] de Oliveira CM, Ferreira JdLM. Overview of cicatricial modulators in glaucoma fistulizing surgery. *Int Ophthalmol*. 2020; 40(10): 2789–2796. 32504309 10.1007/s10792-020-01454-w

[13] Mohajeri A, Amigh S. In the search of active nanocarriers for delivery of mitomycin C drug. *Mater Adv*. 2020; 1(6): 1909–1919.

[14] Sun X, Sun P, Li B, et al. A new drug delivery system for mitomycin C to improve intravesical instillation. *Mater Design*. 2016; 110: 849–857.

[15] Sultana T, Van Hai H, Park M, et al. Controlled release of Mitomycin C from modified cellulose based thermo-gel prevents post-operative de novo peritoneal adhesion. *Carbohydr Polym*. 2020; 229: 115552. 31826495 10.1016/j.carbpol.2019.115552

[16] Kojima S, Sugiyama T, Takai S, et al. Effects of gelatin hydrogel loading mitomycin C on conjunctival scarring in a canine filtration surgery model. *Invest Ophthalmol Vis Sci*. 2015; 56(4): 2601–2605. 25813997 10.1167/iovs.15-16486

[17] Cummings J, Allan L, Smyth JF. Encapsulation of mitomycin C in albumin microspheres markedly alters pharmacokinetics, drug quinone reduction in tumour tissue and antitumour activity: implications for the drugs' in vivo mechanism of action. *Biochem Pharmacol*. 1994; 47(8): 1345–1356. 8185643 10.1016/0006-2952(94)90333-6

[18] Li Y, Lin J, Yang X, et al. Self-assembled nanoparticles based on amphiphilic anticancer drug–phospholipid complex for targeted drug delivery and intracellular dual-controlled release. *ACS Appl Mater Interfaces*. 2015; 7(32): 17573–17581. 26234408 10.1021/acsami.5b05038

[19] Schoenberg ED, Blake DA, Swann FB, et al. Effect of two novel sustained-release drug delivery systems on bleb fibrosis: an in vivo glaucoma drainage device study in a rabbit model. *Transl Vis Sci Technol*. 2015; 4(3): 4. 10.1167/tvst.4.3.4 PMC4451500 26046006

[20] Wu M, Wang S, Wang Y, et al. Targeted delivery of mitomycin C-loaded and LDL-conjugated mesoporous silica nanoparticles for inhibiting the proliferation of pterygium subconjunctival fibroblasts. *Exp Eye Res*. 2020; 197: 108124. 32598971 10.1016/j.exer.2020.108124

[21] Balusamy B, Celebioglu A, Senthamizhan A, Uyar T. Progress in the design and development of “fast-dissolving” electrospun nanofibers based drug delivery systems - a systematic review. *J Control Release*. 2020; 326: 482–509. 32721525 10.1016/j.jconrel.2020.07.038

[22] Xue J, Wu T, Dai Y, Xia Y. Electrospinning and electrospun nanofibers: methods, materials, and applications. *Chem Rev*. 2019; 119(8): 5298–5415. 30916938 10.1021/acs.chemrev.8b00593 PMC6589095

[23] Yoo HS, Kim TG, Park TG. Surface-functionalized electrospun nanofibers for tissue engineering and drug delivery. *Adv Drug Deliv Rev*. 2009; 61(12): 1033–1042. 19643152 10.1016/j.addr.2009.07.007

[24] Drumright RE, Gruber PR, Henton DE. Polylactic acid technology. *Adv Mater*. 2000; 12(23): 1841–1846.

[25] Zhang N, Liu R, Liu X, et al. Personalized 3D-printed amniotic fornical ring for ocular surface reconstruction. *Int J Bioprint*. 2023; 9(3): 713. 37273984 10.18063/ijb.713 PMC10236349

[26] Yan D, Zhang S, Yu F, et al. Insight into levofloxacin loaded biocompatible electrospun scaffolds for their potential as conjunctival substitutes. *Carbohydr Polym*. 2021; 269: 118341. 34294349 10.1016/j.carbpol.2021.118341

[27] Scaffaro R, Maio A, Nostro A. Poly (lactic acid)/carvacrol-based materials: preparation, physicochemical properties, and antimicrobial activity. *Appl Microbiol Biotechnol*. 2020; 104: 1823–1835. 31925482 10.1007/s00253-019-10337-9

[28] Lopes MS, Jardini A, Maciel Filho R. Poly (lactic acid) production for tissue engineering applications. *Procedia Eng*. 2012; 42: 1402–1413.

[29] Liu S, Qin S, He M, et al. Current applications of poly (lactic acid) composites in tissue engineering and drug delivery. *Compos B Eng*. 2020; 199: 108238.

[30] Liang L, Xu XD, Chen CS, et al. Evaluation of the biocompatibility of novel peptide hydrogel in rabbit eye. *J Biomed Mater Res B Appl Biomater*. 2010; 93(2): 324–332. 20225215 10.1002/jbm.b.31562

[31] Ruoslahti E, Pierschbacher MD. New perspectives in cell adhesion: RGD and integrins. *Science*. 1987; 238(4826): 491–497. 2821619 10.1126/science.2821619

[32] Hou Z, Li Y, Huang Y, et al. Phytosomes loaded with mitomycin C–soybean phosphatidylcholine complex developed for drug delivery. *Mol Pharm*. 2013; 10(1): 90–101. 23194396 10.1021/mp300489p

[33] Khajehzadeh M, Sadeghi N. Molecular structure, the effect of solvent on UV–vis and NMR, FT–IR and FT–Raman spectra, NBO, frontier molecular orbital analysis of Mitomycin anticancer drug. *J Mol Liq*. 2018; 256: 238–246.

[34] Garlotta D . A literature review of poly (lactic acid). *J Polym Environ*. 2001; 9: 63–84.

[35] Krishnamurthy V, Kamel IL, Wei Y. Analysis of plasma polymerization of allylamine by FTIR. *J Polym Sci A Polym Chem*. 1989; 27(4): 1211–1224.

[36] Polak-Kraśna K, Abaei AR, Shirazi RN, et al. Physical and mechanical degradation behaviour of semi-crystalline PLLA for bioresorbable stent applications. *J Mech Behav Biomed Mater*. 2021; 118: 104409. 33836301 10.1016/j.jmbbm.2021.104409

[37] Cantor LB, Mantravadi A, WuDunn D, et al. Morphologic classification of filtering blebs after glaucoma filtration surgery: the Indiana Bleb Appearance Grading Scale. *J Glaucoma*. 2003; 12(3): 266–271. 12782847 10.1097/00061198-200306000-00015

[38] Smith M, Chipman ML, Trope GE, Buys YM. Correlation between the Indiana bleb appearance grading scale and intraocular pressure after phacotrabeculectomy. *J Glaucoma*. 2009; 18(3): 217–219. 19295376 10.1097/IJG.0b013e31817d23e0

[39] Hoffmann EM, Herzog D, Wasielica-Poslednik J, et al. Bleb grading by photographs versus bleb grading by slit-lamp examination. *Acta Ophthalmol*. 2020; 98(5): e607–e610. 31889404 10.1111/aos.14335

[40] Wei H, Wang J, Wang R, et al. Effects of atorvastatin on the function of Tenon's capsule fibroblasts in human eyes. *Int Ophthalmol*. 2023; 43(10): 3707–3715. 37422546 10.1007/s10792-023-02780-5

[41] Shu DY, Lovicu FJ. Myofibroblast transdifferentiation: The dark force in ocular wound healing and fibrosis. *Prog Retin Eye Res*. 2017; 60: 44–65. 28807717 10.1016/j.preteyeres.2017.08.001 PMC5600870

[42] Zhang K, Rekhter MD, Gordon D, Phan SH. Myofibroblasts and their role in lung collagen gene expression during pulmonary fibrosis. A combined immunohistochemical and in situ hybridization study. *Am J Pathol*. 1994; 145(1): 114. 7518191 PMC1887314

[43] Wolters J, van Mechelen R, Al Majidi R, et al. History, presence, and future of mitomycin C in glaucoma filtration surgery. *Curr Opin Ophthalmol*. 2021; 32: 148–159. 33315724 10.1097/ICU.0000000000000729

[44] Sun J, Liu X, Lei Y, et al. Sustained subconjunctival delivery of cyclosporine A using thermogelling polymers for glaucoma filtration surgery. *J Mater Chem B*. 2017; 5: 6400–6411. 32264457 10.1039/c7tb01556a

[45] Zhang F, Liu K, Pan Z, et al. Effects of rosiglitazone/PHBV drug delivery system on postoperative fibrosis in rabbit glaucoma filtration surgery model. *Drug Deliv*. 2019; 26: 812–819. 31389267 10.1080/10717544.2019.1648590 PMC6713170

[46] Hollo G . Wound healing and glaucoma surgery: modulating the scarring process with conventional antimetabolites and new molecules. *Dev Ophthalmol*. 2017; 59: 80–89. 28442689 10.1159/000458488

[47] Ma J, Wang L, Dong A, et al. Dynamic regulation of thermal-responsive fibers for mitomycin C-controlled delivery on glaucoma drainage device implantation. *Biomater Adv*. 2025; 176: 214359. 40460687 10.1016/j.bioadv.2025.214359

[48] Gupta R, Yarnall BW, Giuliano EA, et al. Mitomycin C: a promising agent for the treatment of canine corneal scarring. *Vet Ophthalmol*. 2011; 14: 304–312. 21929607 10.1111/j.1463-5224.2011.00877.x PMC3354612

