# Supplementary material for: Inhibiting Postoperative Fibrosis in Glaucoma Filtration Surgery Through Porous PLLA/RGD Membrane Mediated Local/Sustained Delivery of Mitomycin C
Source: Transl Vis Sci Technol. 2025 Dec 1;14(12):1. doi: 10.1167/tvst.14.12.1 (PMC12697704; doi:10.1167/tvst.14.12.1)
Supplement: Supplement 1 [file tvst-14-12-1_s001.docx]

**Inhibiting Postoperative Fibrosis in** **Glaucoma Filtration Surgery through Porous PLLA Membrane Mediated Local/Sustained Delivery of Mitomycin C**

Xia Wu^a^, Kaili Wu^b^, Shibing Ni^c^, Shiyi Song^a^, Dadong Jia^a^, Junjie Tang^d^, Jiabing Ran^e,^ *, Liang Liang^a,^ *

^a^ Department of Ophthalmology, The Second People’s Hospital of China Three Gorges University, The Second People’s Hospital of Yichang, Hubei, China.

^b^ Zhongshan Ophthalmic Center, State Key Laboratory of Ophthalmology, Sun Yat-Sen University, Guangdong Provincial Clinical Research Center for Ocular Diseases, Guangzhou, China

^c^ College of Materials and Chemical Engineering, Key Laboratory of Inorganic Nonmetallic Crystalline and Energy Conversion Materials, China Three Gorges University, Yichang, 443002, China

**^d^** Hubei Institute Geological Prospecting Equipment, Wuhan 430000, China

^e^ College of Biological and Pharmaceutical Sciences, China Three Gorges University, Yichang, 443002, China

^*^Corresponding Authors: Jiabing Ran ([jiabingran@outlook.com](mailto:jiabingran@outlook.com); [ranjiabing@ctgu.edu.cn](mailto:ranjiabing@ctgu.edu.cn)); Liang Liang ([liangliang419519@126.com](mailto:liangliang419519@126.com); [liangliang419519@163.com](mailto:liangliang419519@163.com)).


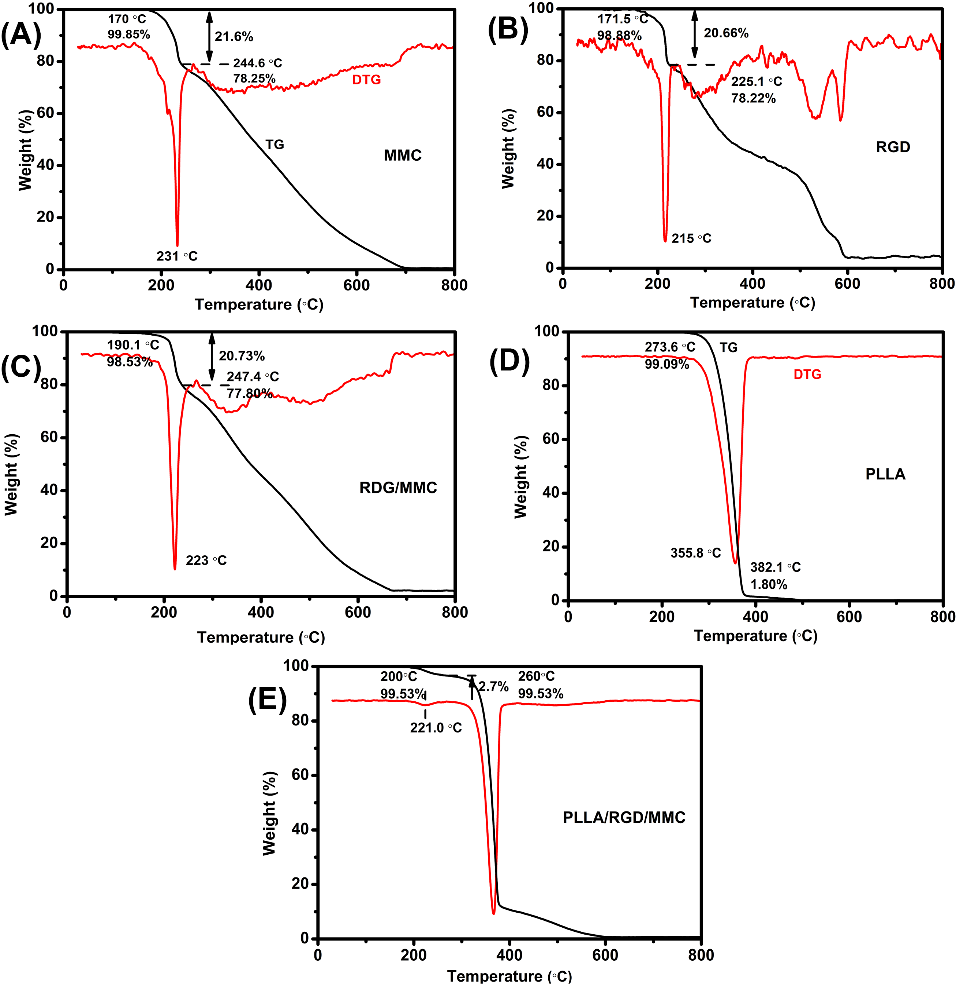
**S1. MMC Content of the As-Prepared PLLA/RGD/MMC ENMs**

**Fig.S1** TG/DTG curves of (A) MMC, (B) dehydrated RGD hydrogel, (C) dehydrated RGD/MMC hydrogel, (D) PLLA membrane, and (E) PLLA/RGD/MMC ENM.

Here, TG was utilized to determine the MMC content of the as-prepared PLLA/RGD/MMC ENMs. We assumed the mass of MMC, PLLA, and RGD were m_MMC,_ m_PLLA,_ and m_RGD,_ respectively. And the mass of the as-prepared PLLA/RGD/MMC ENM was assumed to be m. **Fig.S1** demonstrates the TG and DTG curves of MMC, PLLA, RGD/MMC, PLLA/RGD, and PLLA/RGD/MMC (O_2_ atmosphere, rt to 800 ℃). Obviously, MMC and RGD exhibited thermal degradation within the temperature range between 200 and 300 ℃ while PLLA showed no thermal degradation in this temperature range. As to the RGD/MMC hydrogel**,** we could obtain the following equation from **Fig.S1A-S1C**:

$21.6\%m_{MMC}+20.66\%m_{RGD}=(m_{MMC}+m_{RGD})\times20.73\%$ (1)

Here, we could calculate the mass ratio of m_RGD_/m_MMC_, 12.4286.

Regarding the PPLA/RGD/MMC ENMs, we could obtain the following equation from **Fig.S1A, Fig.S1B,** and **Fig. S1E**:

$21.6\%m_{MMC}+20.66\%m_{RGD}=2.70\%m$ (2)

Combining equation (1) and (2), we could calculate the content of MMC within the PLLA/RGD/MMC ENM.

$$w=\frac{m_{MMC}}{m}=0.97\%$$

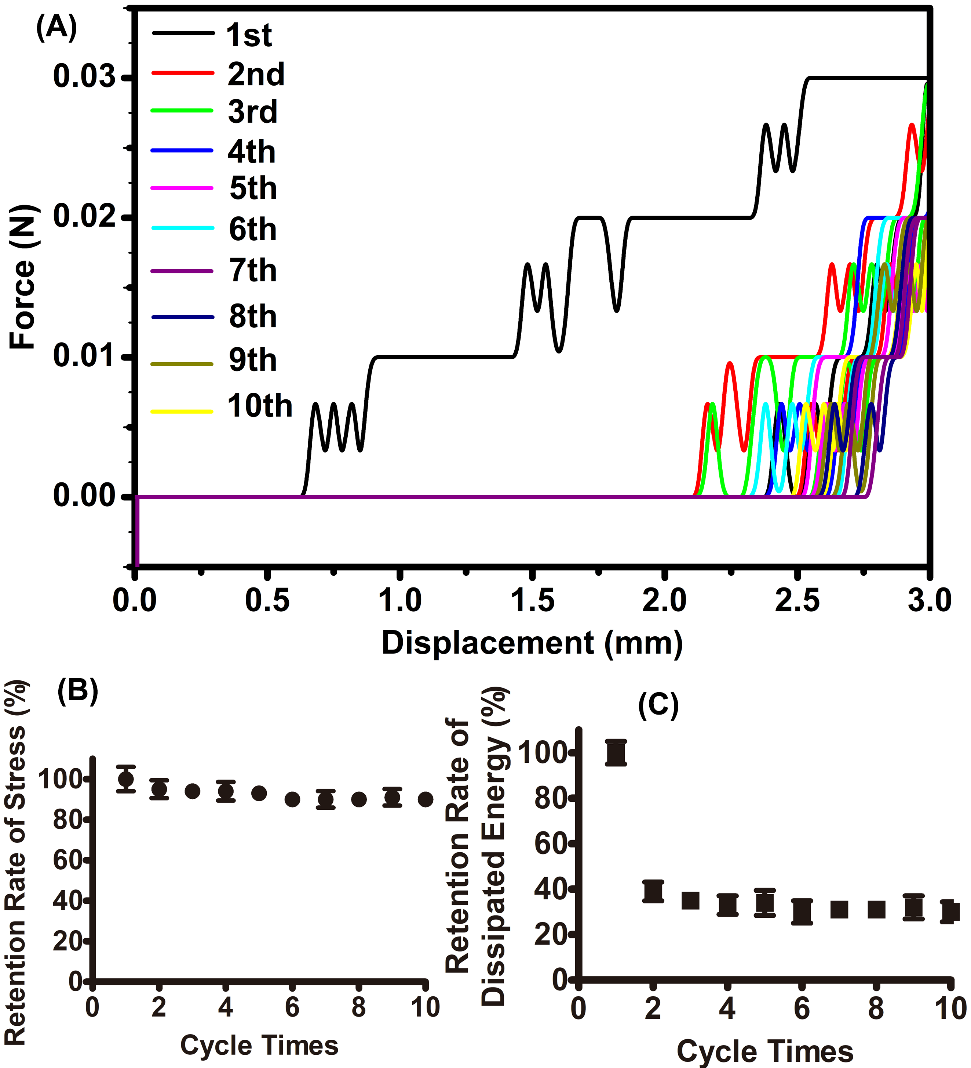


**Fig.S2** (A) Successive load-unloading curves the PLLA/RGD/MMC membrane with strain of 0.1 (10 times); Retention rate of (B) strength and (C) dissipated energy of the ten successive loading-unloading tests.

**
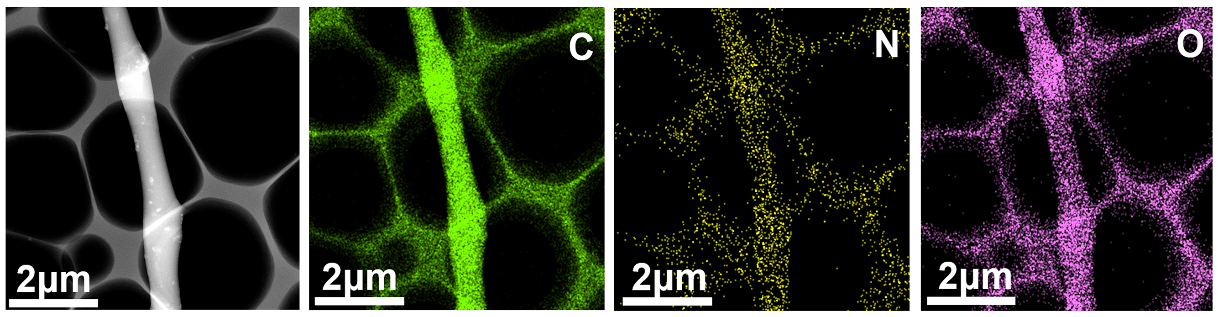
Fig.S3** EDS mapping of as-prepared PLLA/RGD/MMC membrane


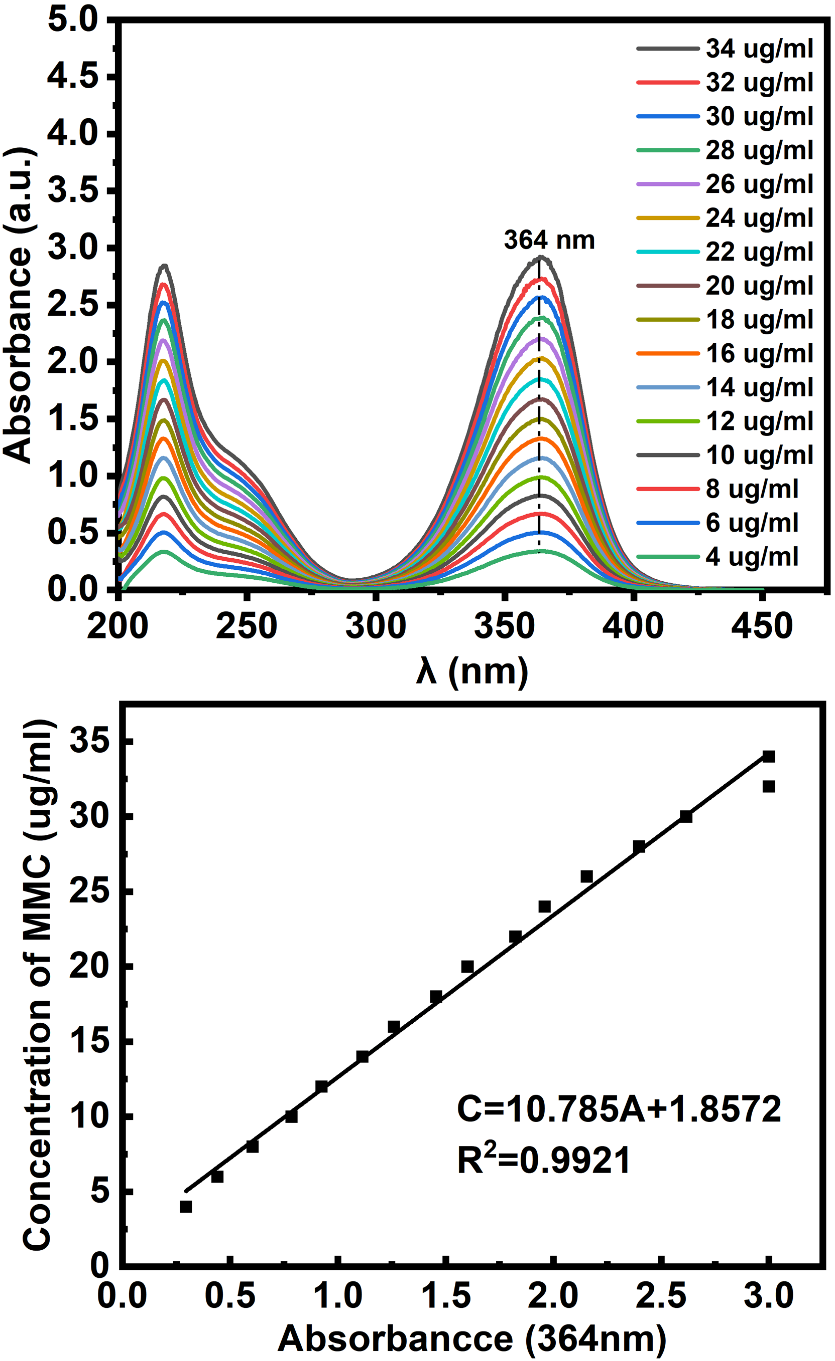


**Fig.S4** (upper) UV-vis spectra of the MMC solutions with gradient concentrations and (lower) fitted standard curve of MMC concentration versus absorbance


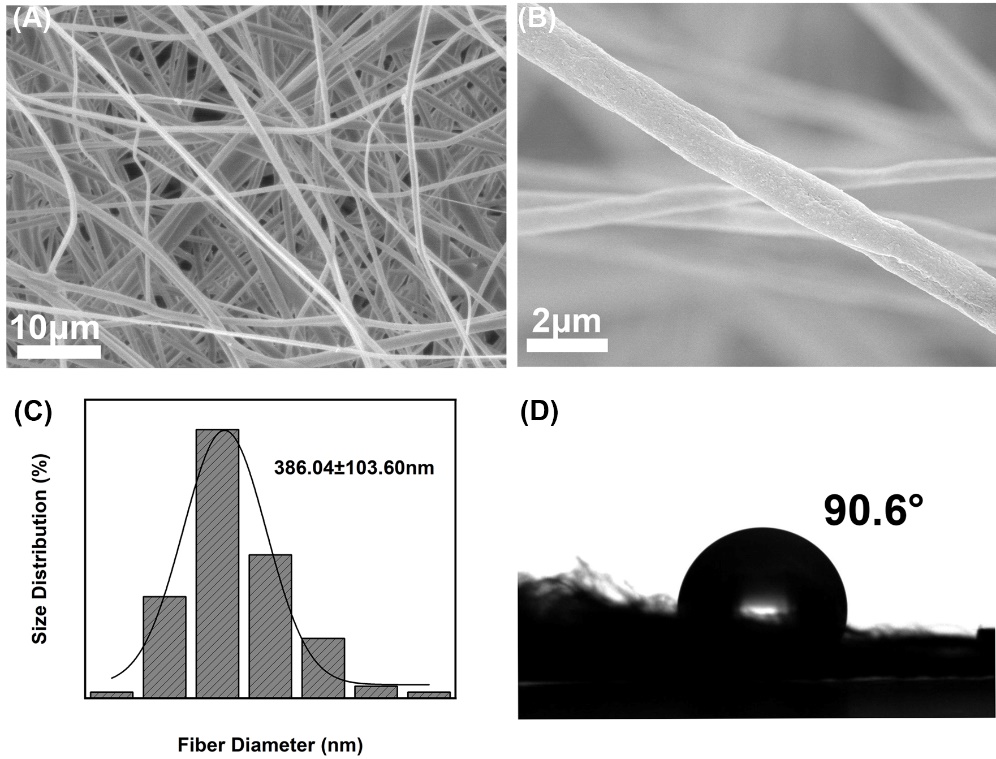
**Fig.S5** (A/B) SEM images of the PLLA/RGD/MMC2 membrane; (C) fiber diameter distribution obtained from (A); Water contact angel of the PLLA/RGD/MMC2 membrane

**S2. Mathematical Modeling and release kinetics [[1](#_ENREF_1), [2](#_ENREF_2)]**

1. Zero-order model

$Q_{t}=Q_{0}+K_{0}t$ (6)

Where Q_t_ is the amount of drug dissolved in time t, Q_0_ is the initial amount of drug in the solution (most times, Q_0_=0) and K_0_ is the zero-order release constant expressed in units of concentration/time.

Application: This relationship is utilized to describe the drug dissolution of several types of modified release pharmaceutical dosage forms, as in the case of some transdermal systems, as well as matrix tablets with low soluble drugs in coated forms, osmotic systems, etc.

1. First-order model

$\log\left( C \right)=\log\left( C_{0} \right)-kt/2.303$ (7)

Where C_0_ is the initial concentration of drug, k is the first order rate constant, and t is the time.

Application: This relationship is used to describe the drug dissolution in pharmaceutical dosages forms such as those containing water-soluble drugs in porous matrices.

1. Higuchi model

$$Q_{t}=Q_{0}+k_{H}t^{\frac{1}{2}} (8)$$

Where Q_t_ is the cumulative amount of drug release at time t, Q_0_ is the initial amount of drug, k_H_ is the Higuchi constant.

Application: This relationship is used to describe the drug release from an insoluble matrix based on Fickian diffusion. It also can be used to describe the drug dissolution from several types of modified release pharmaceutical dosage forms, as in the case of some transdermal systems and matrix tablets with water soluble drugs.

1. Hixson-Crowell model

$W_{0}^{1/3}-W_{t}^{\frac{1}{3}}=\kappa t$(9)

Where W_0_ is the initial amount of drug in the pharmaceutical dosage form, W_t_ is the remaining amount of drug in the pharmaceutical dosage form at time t and κ is a constant incorporating the surface-volume relation.

Application: This expression applies to pharmaceutical dosage form such as tablets, where the dissolution occurs in planes that are parallel to the drug surface if the tablet dimensions diminish proportionally, in such a manner that the initial geometrical form keeps constant all the time.

1. Korsmeyer-Peppas model

$Q_{t}=K_{kp}t^{n}$ (10)

Where Q_t_ is the cumulative amount of drug release at time t, K_kp_ is the Korsmeyer-Peppas constant, n is the release exponent which defines the drug release mechanism.

Application: This relationship is used to describe drug release from the polymeric system. When the Korsmeyer-Peppas model is used to thin films, the release index n (n = 0.5 or n ≤ 0.5) conforms to Fickian diffusion, while the values of n (0.5＜n＜1) is related to non-Fickian release, meaning that the drug release follows both erosion and diffusion mechanisms. And n = 1 corresponds to the zero-order release which defines the drug release is independent of time.

1. Kopcha model

$Q_{t}=At^{1/2}+Bt$ (11)

Where A is the diffusion constant and B is the erosional exponent.

Application: If the ratio of A/B is high (A is much greater than B), implying the drug release will follow the diffusion mechanism; and if A/B is low (B is much greater than A), meaning that the polymer erosion or relaxation plays a dominant role in drug release.

1. Weibull model

$M=M_{0}[1-e^{-\frac{{(t-T)}^{b}}{a}}$] (12)

Where M is the amount of drug dissolved as a function of time t. M_0_ is total amount of drug being released. T accounts for the lag time measured as a result of the dissolution process. Parameter a denotes a scale parameter that describes the time dependence, while b describes the shape pf the dissolution curve progression.

Application: The Weibull model is more useful for comparing the release profiles of matrix type drug delivery.


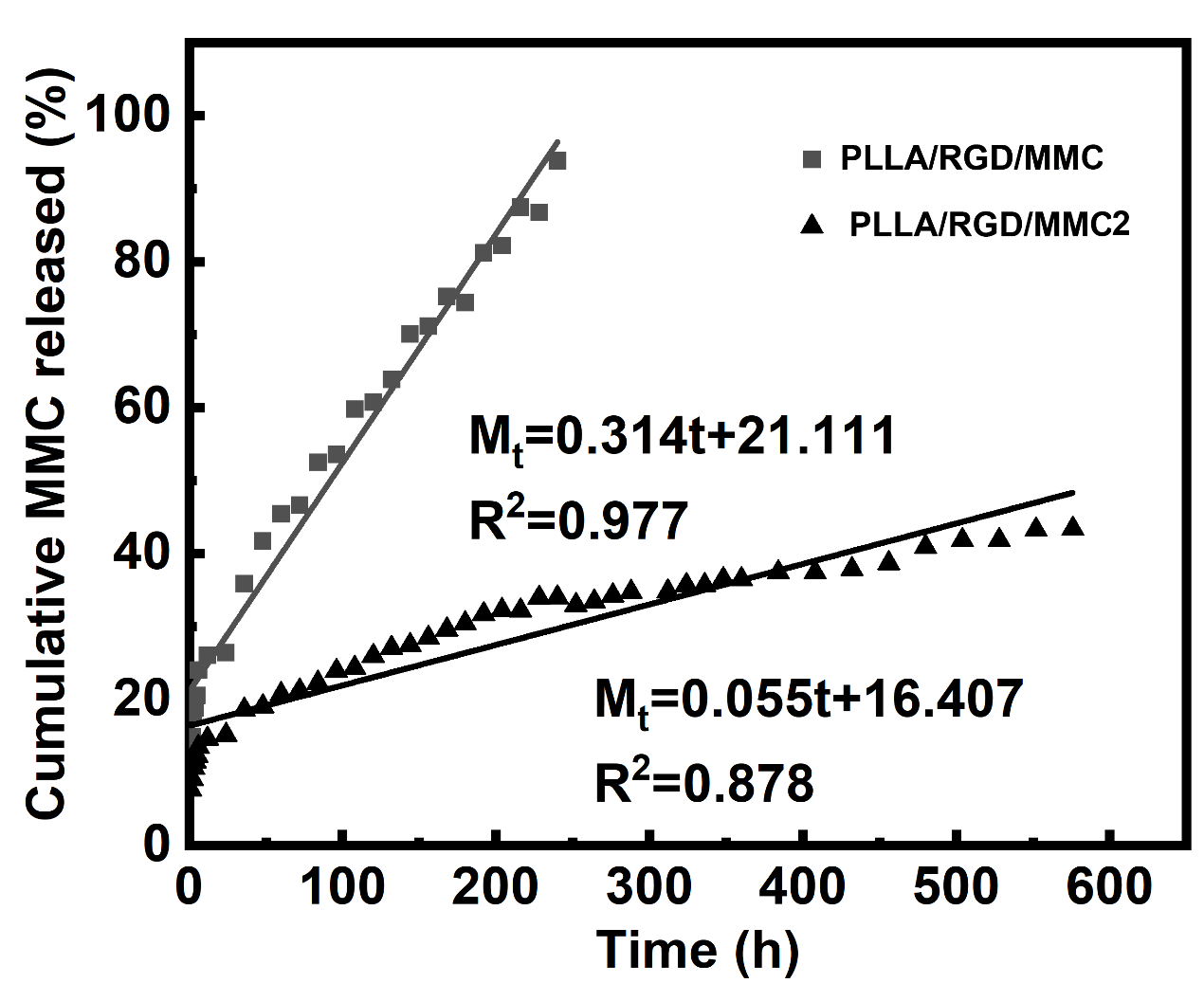


**Fig.S6** Fitted curves with Zero-order model


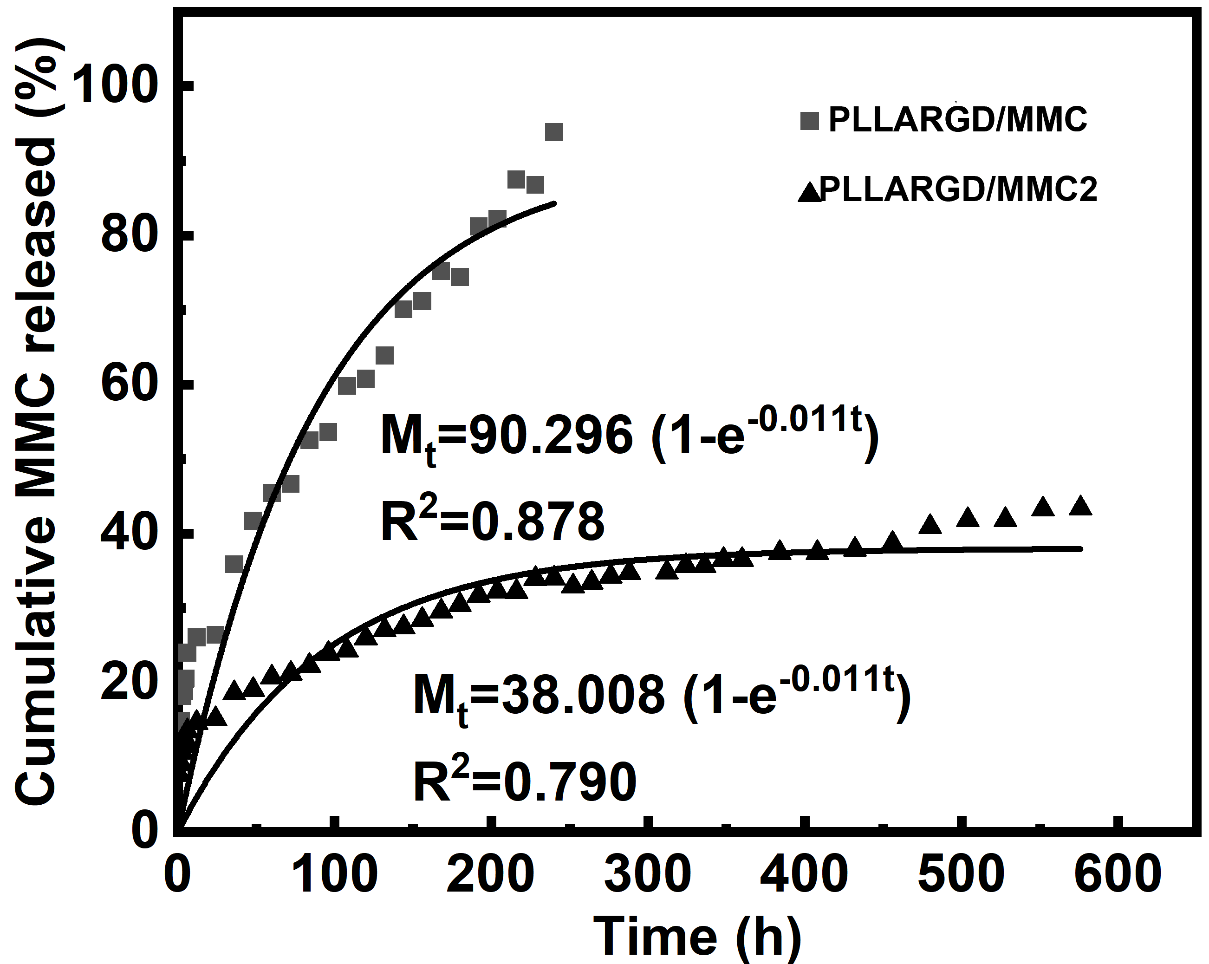


**Fig.S7** Fitted curves with First-order model


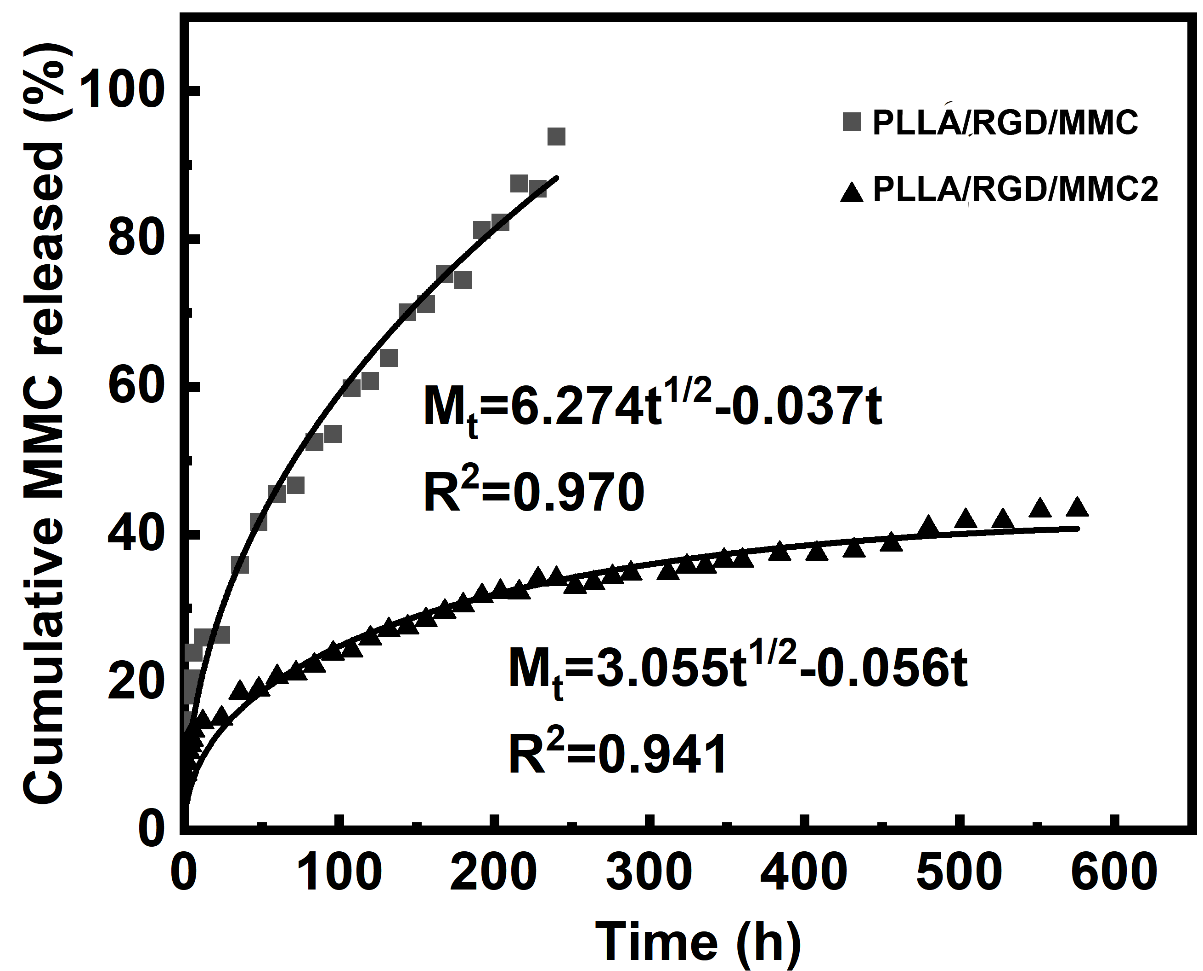


**Fig.S8** Fitted curves with Kopcha model


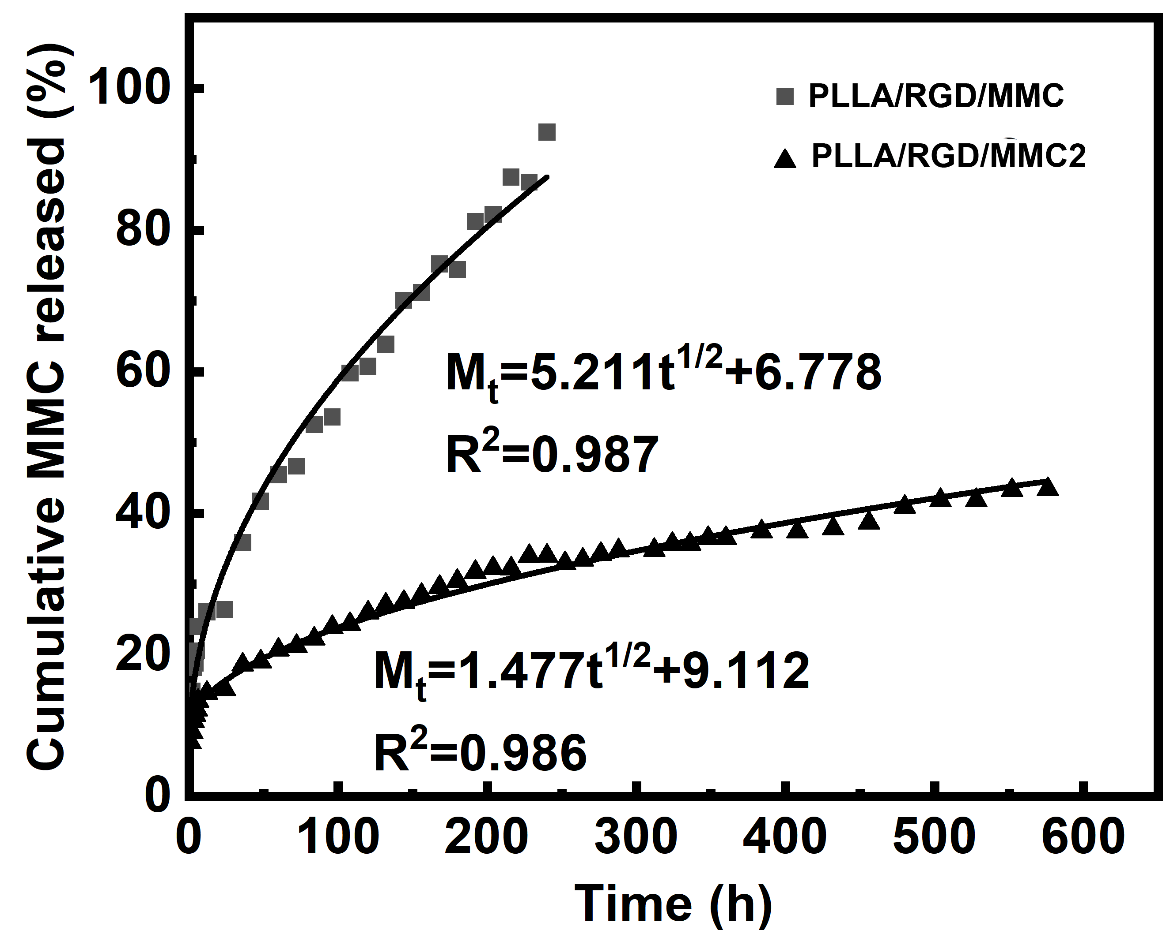


**Fig.S9** Fitted curves with Higuchi model

**References**

[1] Dash S, Murthy PN, Nath L, Chowdhury P. Kinetic modeling on drug release from controlled drug delivery systems. Acta Pol Pharm 2010;67:217-23.

[2] Xiang Z, Liu T, Wang H, Chen G, Zhu X, Hao T, et al. Rational design of a supramolecular hydrogel with customizable pH-responsiveness on the basis of pH-induced ionization/protonation transition of BSA. Soft Matter 2022;18:3157-67.
